# Genome-Wide Analysis of WUSCHEL-Related Homeobox Gene Family in Sacred Lotus (*Nelumbo nucifera*)

**DOI:** 10.3390/ijms241814216

**Published:** 2023-09-18

**Authors:** Gui-Zhen Chen, Jie Huang, Zhi-Cong Lin, Fei Wang, Song-Min Yang, Xiao Jiang, Sagheer Ahmad, Yu-Zhen Zhou, Siren Lan, Zhong-Jian Liu, Dong-Hui Peng

**Affiliations:** Key Laboratory of National Forestry and Grassland Administration for Orchid Conservation and Utilization, College of Landscape Architecture, Fujian Agriculture and Forestry University, Fuzhou 350002, China; cgz1020@126.com (G.-Z.C.); 13530401396@163.com (J.H.); hakunamatata0826@163.com (Z.-C.L.); wangfei_icon@163.com (F.W.); songminyang3199@163.com (S.-M.Y.); jxfafu996@163.com (X.J.); sagheerhortii@gmail.com (S.A.); zhouyuzhencn@163.com (Y.-Z.Z.); lkzx@fafu.edu.cn (S.L.)

**Keywords:** *NnWOXs*, transcription factor, *Nelumbo nucifera*, protein subcellular localization, gene expression, gene network

## Abstract

WUSCHEL-related homeobox (WOX) is a plant-specific transcription factor (TF), which plays an essential role in the regulation of plant growth, development, and abiotic stress responses. However, little information is available on the specific roles of WOX TFs in sacred lotus (*Nelumbo nucifera*), which is a perennial aquatic plant with important edible, ornamental, and medicinal values. We identified 15 WOX TFs distributing on six chromosomes in the genome of *N. nucifera*. A total of 72 WOX genes from five species were divided into three clades and nine subclades based on the phylogenetic tree. *NnWOXs* in the same subclades had similar gene structures and conserved motifs. Cis-acting element analysis of the promoter regions of *NnWOXs* found many elements enriched in hormone induction, stress responses, and light responses, indicating their roles in growth and development. The Ka/Ks analysis showed that the WOX gene family had been intensely purified and selected in *N. nucifera*. The expression pattern analysis suggested that *NnWOXs* were involved in organ development and differentiation of *N. nucifera*. Furthermore, the protein–protein interaction analysis showed that *NnWOXs* might participate in the growth, development, and metabolic regulation of *N. nucifera*. Taken together, these findings laid a foundation for further analysis of *NnWOX* functions.

## 1. Introduction

Transcription factor (TF) is a DNA-binding protein that can specifically bind to cis-acting elements in the promoter and regulate plant growth and development, and response to stress [1]. The WOX gene family belongs to the Homeobox (HOX) super family, which plays a vital role in plant growth, development, and abiotic stress responses [2,3,4,5,6]. A total of 15 *AtWOXs* are found in *Arabidopsis thaliana*, including *AtWUS* and *AtWOX1~AtWOX14* [7,8,9]. Phylogenetic tree analysis shows that the WOX gene family of *A. thaliana* can be divided into three clades: WUS, intermediate, and ancient [10].

Recent functional studies of the WOX gene family show that it is significantly involved in the regulation of stem cell division and differentiation, embryo and organ formation, and flower development [11,12,13,14,15]. In *A. thaliana*, *AtWUS* induces the stem tip to form apical meristem, maintains the activity of apical meristem, affects the development of ovary and anther, and controls the development of leaves and inflorescences [11,13,14]. *AtWOX1* regulates the transverse growth of leaves and affects its width [4]. *AtWOX3* is involved in the development of petals [16]. *AtWOX5* has a function similar to that of *AtWUS* and regulates the expression of apical meristem [17]. *AtWOX11* and *AtWOX12* are involved in the development of explant roots [18,19], while *AtWOX13* and *AtWOX14* control root and flower development [20]. In rice, *OsWUS* is specifically expressed in root apical meristem [21]. *OsWOX3* is a homologous gene of *AtWOX3*, which is expressed in the primordia of leaves and floral organs, and plays an import role in the development of leaves [22]. *OsWOX9* is a homologous gene of *AtWOX5*, and regulates the activity of apical meristem [22]. In addition, studies have shown that WOX transcription factor has a key role in plant response to abiotic stresses such as drought, low temperature and salt stress [23]. In the *HOS9-1* mutant of *Arabidopsis*, *AtWOX6* can affect the response to cold stress [24]. In rice, *OsWOX11* enhances drought resistance by regulating the development of root hairs [25]. Furthermore, WOX genes can also interact with hormones to regulate plant growth and development [26,27,28,29].

*Nelumbo*, Nelumbonaceae, is a basal eudicot with important value in terms of understanding the origin and evolution of eudicots. *Nelumbo* species are perennial aquatic herbs with high ornamental values. This genus consists of two species: Asian lotus (*N. nucifera* Gaertn.) and American lotus (*N. lutea* Willd.) [30]. *N. nucifera* is mainly distributed in China, India, Korean Peninsula, Japan, Vietnam, Thailand, and Australia [31]. Due to its rich and diverse color and flower patterns, *N. nucifera* is widely used in the landscape, and also has edible and medicinal values [30]. The WOX gene family has been identified in many plants, such as *A. thaliana* [11], *Selaginella kraussiana* [10], rice [21], cotton [5], sorghum [6], maize [6], and poplar [6]. Furthermore, the WOX gene family plays an important regulatory role in flower development. However, there is a lack of studies in *N. nucifera*. Given lotus’s importance and WOX genes’ significant effect on flower development, we make a comprehensive understanding of the WOX gene family in *N. nucifera*.

The aim of this study was to fill the gap of the WOX gene family in *N. nucifera* research, making an understanding of the regulatory mechanism of the *N. nucifera* flower development. Our genome-wide expression-profiling analysis of the WOX TFs in lotus will provide a fundamental platform for candidate gene selection in flower development, providing valuable information for future studies on the functions of the candidate gene. A total of 15 WOX genes were identified in the whole genome of *N. nucifera.* This is the first report on the physicochemical properties, phylogenetic relationships, protein structures, cis-acting regulatory elements in promoters, chromosomal distribution, and duplication events of *NnWOX* genes in the *N. nucifera* genome. Gene expression analysis from available transcriptome data of the *N. nucifera* genome revealed the significant expression of *NnWOX* TFs in shoot and floral organs of *N. nucifera*. Taken together, this study provides the involvement of WOX gene family during growth and development in *N. nucifera*.

## 2. Results

### 2.1. Identification of WOX Genes and the Analysis of Physicochemical Properties

The WOX genes of *A. thaliana* and *O. sativa* were used as the query sequences to identify the WOX genes in *N. nucifera* and *Ny. colorata*. A total of 15 WOX genes were identified each in *N. nucifera* and *Ny. colorata*. According to the location of the WOX genes on the chromosome, the 15 *NnWOXs* were named *NnWOX1-NnWOX15* (Supplementary Figure S1). The analysis of physicochemical properties of WOX proteins showed significant differences among *NnWOXs* (Table 1). The length of amino acid was between 182~366 aa, and the molecular weight was between 20,640.22 (*NnWOX6*) and 40,294.14 Da (*NnWOX4*). The theoretical isoelectric point (PI) was between 5.42 and 9.46; *NnWOX2* showed the highest PI and *NnWOX10* showed the lowest PI. There were eight proteins with PI more significant than 7, which were essential proteins, and the other seven proteins were acidic proteins. The aliphatic index NnWOX proteins ranged between 51.47 and 71.65, suggesting that all of them were unstable. The grand average of hydropathicity (GRAVY) was between −0.935 and −0.289, indicating that the WOX proteins were hydrophilic, and the degree of hydropathicity was different for each protein. The difference in the various physical and chemical properties of NnWOX proteins indicates their functional diversity. Prediction of the subcellular localization showed that all NnWOX genes were located in the nuclear region.

### 2.2. Phylogenetic Relationships of WOXs

**Figure 1:**
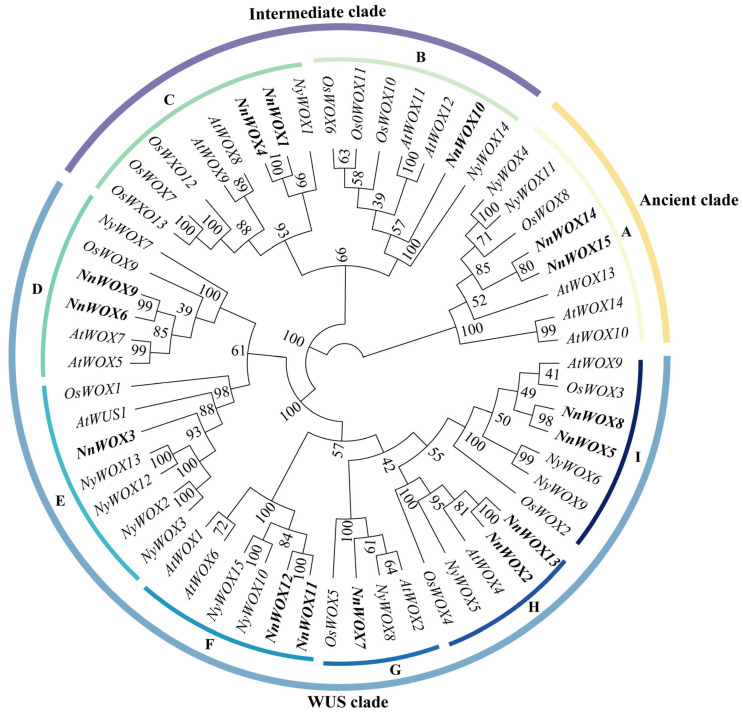
Phylogenomic tree of WOX gene family. The bold content is the WOX genes in *N. nucifera.* The genes begin with “At” to represent the genes of *A. thaliana*, “Nn” to represent the genes of *N. nucifera*, “Nc” to represent the genes of *Ny. colorata*, “Pe” to represent the genes of *P. equestris*, and “Os” to represent the genes of *O. sativa*. (A–I) represent subclades (A–I).

The phylogenetic tree was constructed from the WOX gene families of *A. thaliana* (15), *O. sativa* (14), *N. nucifera* (15), *P. equestris* (13), and *Ny. colorata* (15). The results showed that the 72 WOX genes from five species could be divided into three clades: WUS, ancient, and intermediate (Figure 1). The result showed that the WOX proteins from the five species were unevenly distributed in the three clades. The WUS clade had the most WOX proteins at 42, followed by intermediate clade at 18, and ancient clade had the least at 12. The ancient clade contained 12 WOXs, including three, two, one, four, and two for *A. thaliana*, *N. nucifera*, *O. sativa*, *P. equestris*, and *Ny. colorata*, respectively. The intermediate clade was further divided into two subclades: B and C. The WUS clade was further classified into six subclades: D, E, F, G, H, and I. However, we did not find any subclade H member in *O. sativa* and *P. equestris*.

### 2.3. Protein Structure Analysis

**Figure 2:**
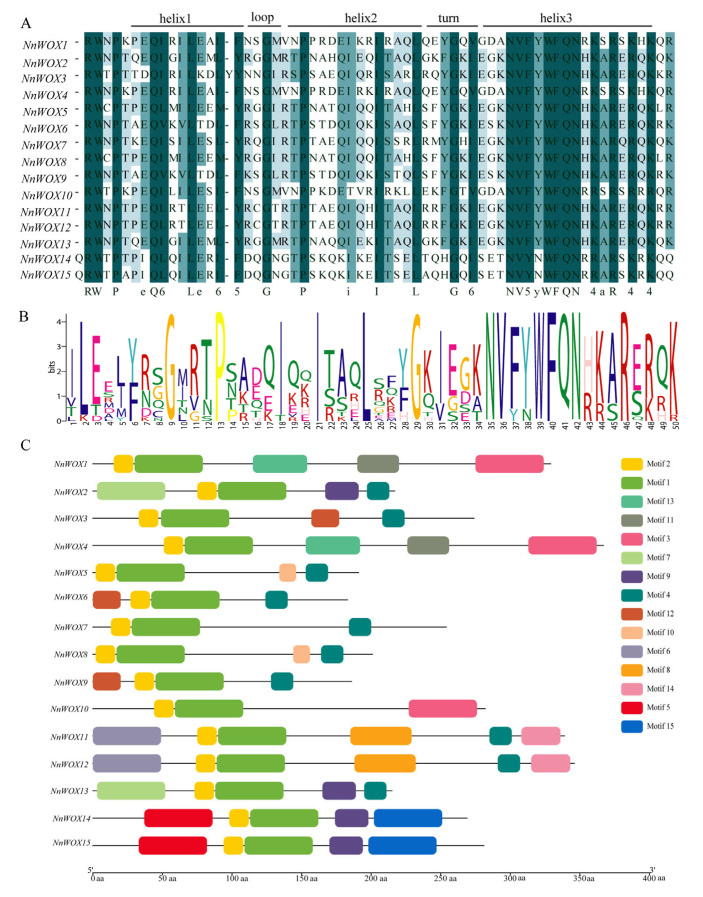
The protein features of WOX gene family in *N. nucifera*. (**A**) The conserved domain of NnWOXs; (**B**) the Seqlogo of NnWOXs; (**C**) the motifs of NnWOX proteins.

The protein sequence alighnment of NnWOXs showed that all the WOX members contained a conserved homo-domain (Figure 2A). The homo-domain contained several conserved amino acids, such as E, Q, L, and E in helix 1; G in loop; P, I, I, and L in helix 2; G in turn; N, V, Y, W, F, Q, N, A, and R in helix 3 (Figure 2A,B). This result is in line with the conserved amino acid residues identified by Zhang et al. [6]. In addition, we also observed additional conserved residues, I in the turn, and R, W, and P before helix 1 among NnWOX proteins. Furthermore, compared with the typical type of homo-domain, an extra amino acid was found in the helix 1 of *NnWOX3.* These results suggested that the homo-domain of WOX proteins was highly conserved in *N. nucifera*.

Using MEME to analyze the conserved motifs of WOX proteins, 15 motifs (1–15) were found (Figure 2C, Supplementary Table S2). Motifs 1 and 2 were highly conserved, which were incorporated into the 15 *NnWOXs*. Three *NnWOXs* had motif 3 and ten *NnWOXs* included motif 4. *NnWOX14* and *NnWOX15* contained motifs 5 and 15. *NnWOX11* and *NnWOX12* contained motifs 6, 7, and 14. *NnWOX2* and *NnWOX3* had motif 7. Four *NnWOXs* contained motif 9. *NnWOX5* and *NnWOX8* contained motif 10. *NnWOX1* and *NnWOX4* had motifs 11 and 13. Three *NnWOXs* contained motif 12. The *NnWOXs* with the same motif were grouped into one clade in the phylogenetic tree (Figure 1).

**Figure 3:**
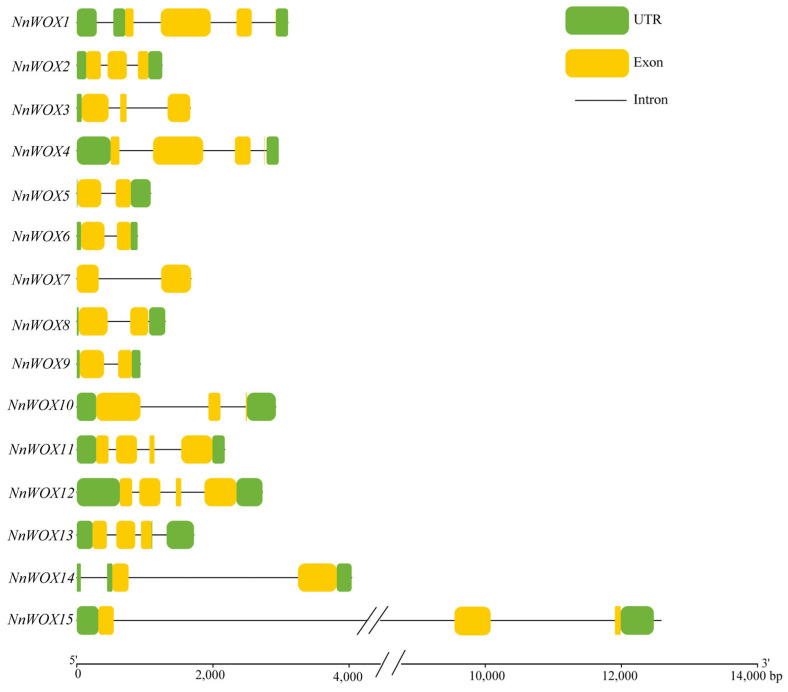
The gene structure of WOX gene family in *N. nucifera*.

The gene structure analysis showed that all *NnWOXs* had introns, the number of which varied from 1 to 3, and the number of exons varied from 2 to 4 (Figure 3). *NnWOX7* did not contain UTR, *NnWOX3* only contained 5′-UTR, and the remaining NnWOXs contained 5′-UTR and 3′-UTR. *NnWOX5*, *NnWOX6*, *NnWOX7*, *NnWOX8*, *NnWOX9*, and *NnWOX14* contained two exons, accounting for 40% of the total NnWOXs. *NnWOX1*, *NnWOX2*, *NnWOX3*, *NnWOX10*, *NnWOX13*, and *NnWOX15* contained two exons, which accounted for 40% of the total NnWOXs. *NnWOX4*, *NnWOX11*, and *NnWOX13* contained four exons, representing 20% of the total NnWOXs. The NnWOXs with similar gene structure and a close genetic relationship were grouped into one clade in the phylogenetic tree.

### 2.4. Cis-Acting Regulatory Elements Analysis

**Figure 4:**
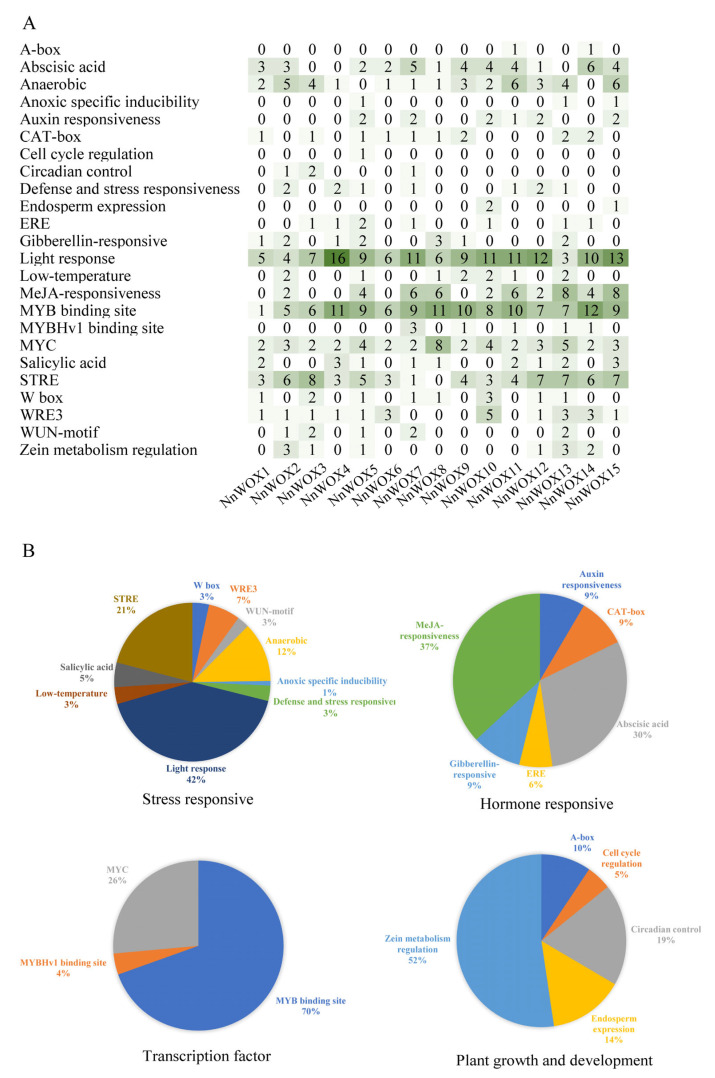
The cis-acting regulatory elements of WOX gene family. (**A**) Cis-elements in the promoters of *NnWOXs*, the degree of green color and the numbers on the grid refer to the number of cis-elements of the *NnWOXs*; (**B**) the percentage of each cis-acting element.

The cis-acting regulatory elements in the promoter region of of *NnWOXs* were analyzed by PlantCARE. Four functional categories of cis-regulatory elements were found, including stress-responsive, plant growth and development, transcription factor, and hormone-responsive elements (Figure 4). The cis-acting elements related to hormones were responsive to auxin, gibberellin, MeJA, and abscisic acid, which play an essential role in the adaptation of plants during environmental stress. The cis-acting elements related to stress included W box (3%), WRE3 (7%), WUN-motif (3%), anaerobic (12%), anoxic specific inducibility (1%), light response (42%), salicylic acid (5%), STRE (21%), defense and stress-responsive (3%), low-temperature-responsive (3%), and anaerobic induction elements, which indirectly affect growth and development. The plant growth and development-related elements included cell cycle regulation (5%), A-box (10%), circadian control (19%), zein metabolism regulation (52%), and endosperm expression (14%). Therefore, *NnWOXs* may be involved in the meristem growth, response to stress, and hormone regulation of *N. nucifera*.

### 2.5. Chromosome Location and Collinearity Analysis

**Figure 5:**
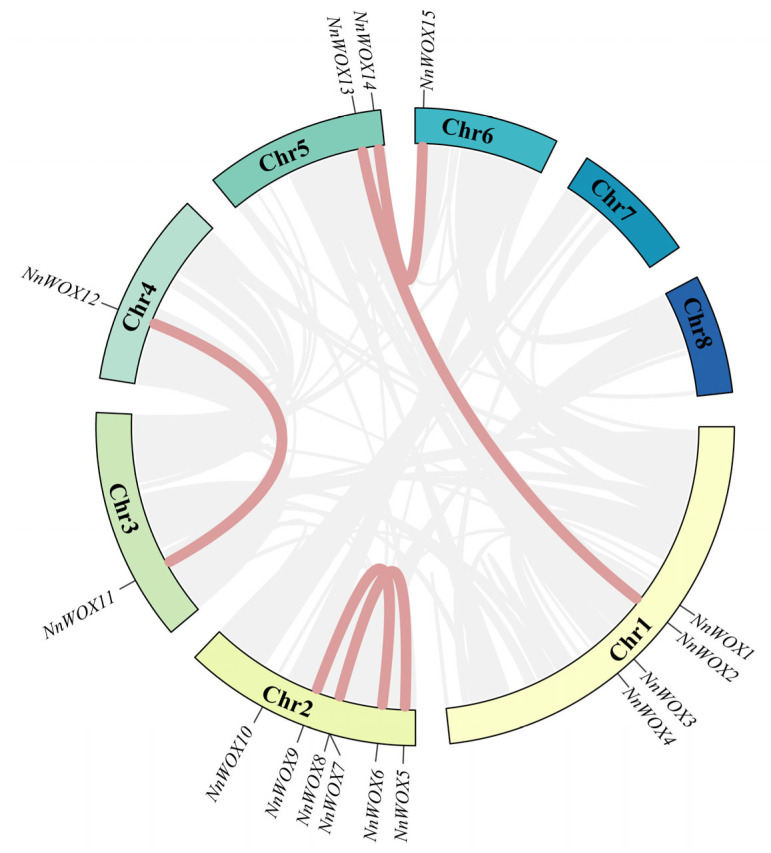
Synteny analysis of *NnWOXs* in *N. nucifera*. The red lines represent the syntenic gene pairs of the WOX genes of *N. nucifera*, and grey lines represent the syntenic gene pairs of *N. nucifera*.

To further analyze the evolutionary relationship among the *NnWOXs*, their chromosome locations were ascertained based on the annotated information of the genome of *N. nucifera*. The results showed that the WOX gene family was not evenly distributed on eight chromosomes of *N. nucifera* genome (Figure 5 and Supplementary Figure S1). The WOX genes were distributed on chromosomes 1, 2, 3, 4, 5 and 6, but no WOX gene was detected on chromosomes 7 and 8. The distribution of WOX genes was the most abundant on chromosome 2, containing six *NnWOXs* (*NnWOX5*-*NnWOX10*), followed by chromosome 1, which contained four *NnWOXs* (*NnWOX1*-*NnWOX4*). Chromosome 5 contained two *NnWOXs* (*NnWOX11* and *NnWOX14*) and chromosomes 3, 4, and 6 only had one *NnWOX*. To further investigate the expansion mechanism of *NnWOX* genes, we analyzed the duplication events by Tbtools and found five pairs of segmental duplication events in the WOX gene family of *N. nucifera* (Figure 5). These segmental duplication genes were not evenly distributed on eight chromosomes. Four duplication events were observed on chromosome 2 and two were distributed on chromosome 5. Each of the chromosomes 1, 3, 4, and 6 exhibited one duplication event. There was no segmental duplication gene on chromosomes 7 and 8. The Ka (non-synonymous substitutions per site), Ks (synonymous substitutions per site), and Ka/Ks (evolutionary constraint) values were calculated for the selection presurre analysis. The resluts showed that Ka/Ks values were all less than 1 (Table 2), indicating that the *N. nucifera* WOX gene family had been intensely purified and selected in the course of evolution.

**Figure 6:**
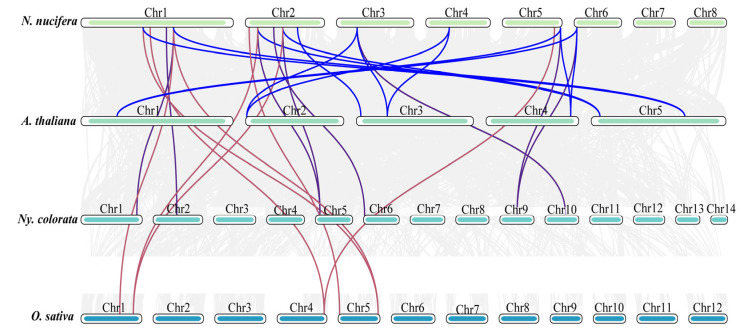
Synteny analysis of WOX gene family between *N. nucifera*, *A. thaliana*, *O. sativa*, and *Ny. colorata*. The blue lines represent the colinear WOX gene pairs between *N. nucifera* with *A. thaliana*; the purple lines represent the colinear WOX gene pairs between *N. nucifera* with *Ny. colorata*; the purple lines represent the colinear WOX gene pairs between *N. nucifera* with *Ny. colorata;* the red lines represent the colinear WOX gene pairs between *N. nucifera* with *O. sativa*; the grey lines represent the colinear gene pairs between *N. nucifera* with *A. thaliana*, *O. sativa*, and *Ny. colorata*.

To further explore the evolutionary mechanism of *NnWOX* TFs, a collinear analysis was carried out between *N. nucifera* and three representative species, namely *A. thaliana*, *O. sativa*, and *Ny. colorata* (Figure 6). The results showed 13 pairs of collinear homologous genes between *N. nucifera* and *A. thaliana*; eight pairs of collinear genes between *N. nucifera* and *Ny. colorata*; and eight pairs between *N. nucifera* and *O. sativa*. The *NnWOX4*, *NnWOX6*, and *NnWOX9* had homologous genes in all three species, suggesting that these three genes may have existed before the differentiation of Angiospermae, and played an important role in these species after differentiation. In addition, the WOX gene family had the best homology with *Ny. colorata*, and these WOX genes, suggesting their divergence from a common ancestor.

### 2.6. Gene Expression Analysis

**Figure 7:**
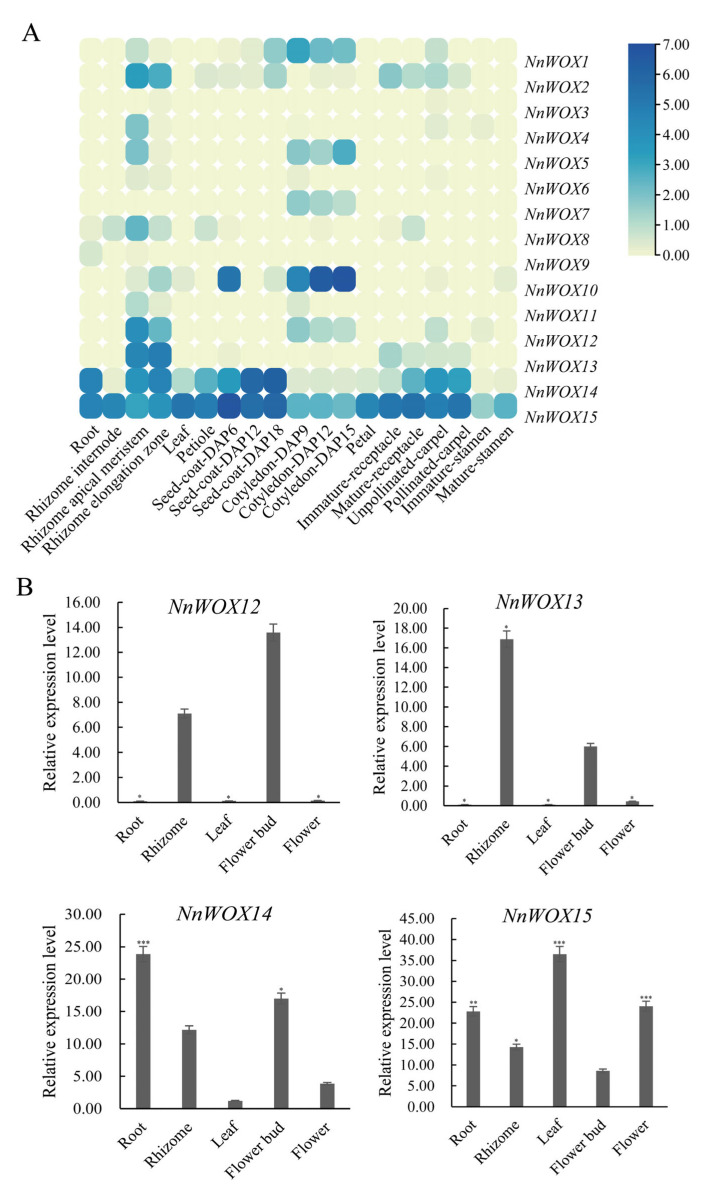
The expression pattern of *NnWOX* gene family in *N. nucifera*. (**A**) The heat map of *NnWOXs* in different organs of *N. nucifera*. The color scale on the right side of the heatmap represents the relative expression level of *NnWOX* genes, and the expression was increased with the color gradient from faint yellow to dark blue; (**B**) the qPCR of *N. nucifera*. The *** represent the significance levels is *p* ≤ 0.001, ** represent the significance levels is *p* ≤ 0.01, and the * represent the significance levels is *p* ≤ 0.05.

Tissue-specific expression analysis showed some differences in the expression of 15 *NnWOXs* in different tissues (Figure 7A). *NnWOX15* was highly expressed in all tissues. *NnWOX14* was highly expressed in root, seed, carpel, leaf, petiole, and receptacle. *NnWOX10* showed significant expression in the cotyledon. *NnWOX13* demonstrated elevated expression in the rhizome, and normal expression in carpel and receptacle. *NnWOX11* was highly expressed in the rhizome, and showed medium expression in the cotyledon. *NnWOX1*, *NnWOX5*, and *NnWOX7* showed moderate expression in the cotyledon. The above results suggest that the *NnWOX* gene family may be involved in different growth and development processes of *N. nucifera.*

To further determine the function of *NnWOX* genes, the tissue-specific expression of *NnWOX* genes was evaluated (Figure 7B). The qRT-PCR result showed that most *NnWOX* genes were expressed in multiple tissues. *NnWOX14* and *NnWOX15* were constitutively expressed in root, rhizome, leaf, flower bud, and booming flower, suggesting that they were involved in multiple developmental stages of *N. nucifera*. The expression levels of *NnWOX12* and *NnWOX13* were high in the rhizome and flower bud, while they did not express in root, leaf, and booming flower. *NnWOX14* had the highest expression in the root and relatively less expression in the leaf. *NnWOX15* showed significant exrpression in leaf, and medium expression in root and flower.

### 2.7. GO Annotation Analysis

GO annotation analysis of the NnWOX proteins showed significant enrichment in ‘cellular component’, ‘molecular function’, and ‘biological process’ (Supplementary Figure S2). In terms of ‘cellular component’ and ‘molecular function’, most members of *NnWOX* TFs were related to DNA binding and the regulation of transcriptional activity in the nucleus. In ‘biological process’, the annotation information of *NnWOX* TFs was diversified. For example, the *NnWOX3* was specially enriched in ‘anther development’, the *NnWOX8* was specially enriched in ‘shoot system development’, and the *NnWOX13* was enriched in ‘procambium histogenesis’.

### 2.8. Protein Interaction Analysis

**Figure 8:**
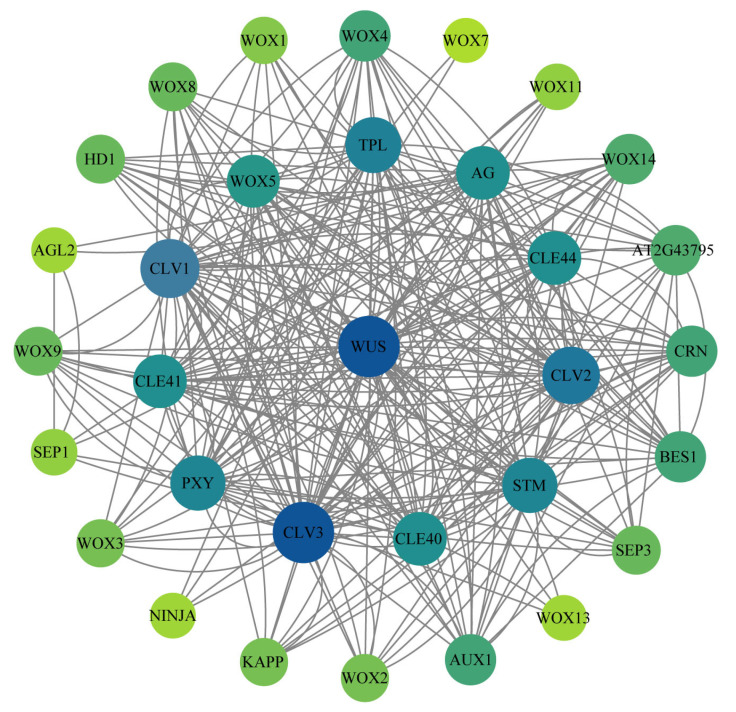
The network of the WOX gene family. The lines present the interaction of proteins. The yellow-green to dark-blue present the importance of the protein in the interaction network. The darker the color, the more important the protein is in the interaction network. *WUS* homologous to *NnWOX3*, WOX1 homologous to *NnWOX11* and *NnWOX12*, WOX2 homologous to *NnWOX7*, WOX3 homologous to *NnWOX5* and *NnWOX8*, WOX4 homologous to *NnWOX2* and *NnWOX13*, WOX5 homologous to *NnWOX6*, *WOX7* homologous to *NnWOX9*, WOX8 homologous to *NnWOX1*, WOX9 homologous to *NnWOX4*, WOX11 homologous to *NnWOX10*, WOX13 homologous to *NnWOX14*, WOX14 homologous to *NnWOX15*.

This study predicted the function of corresponding homologous genes in *N. nucifera* through protein–protein interaction (PPI) analysis of the WOX genes in *A. thaliana*. The interaction protein network of WOX genes was predicted in the String protein interaction database. The results revealed that 20 proteins in the WOX gene family were predicted to interact (Figure 8). Most WOX proteins formed a complex network structure, such as WOX5 (homologous to *NnWOX6*), *WUS* (homologous to *NnWOX3*, with the highest connectivity), WOX4 (homologous to *NnWOX2* and *NnWOX13*), WOX8 (homologous to *NnWOX1*), WOX9 (homologous to *NnWOX4*), WOX3 (homologous to *NnWOX5* and *NnWOX8*), WOX2 (homologous to *NnWOX7*), WOX1 (homologous to *NnWOX11* and *NnWOX12*), WOX14 (homologous to *NnWOX15*), WOX11 (homologous to *NnWOX10*), etc. There were also some WOX proteins that had a simple interaction, such as the *WOX7* (homologous to *NnWOX9*, demonstrated the lowest connectivity) and WOX13 (homologous to *NnWOX14*, with four interactions). These interaction proteins provide insights into the functions and mechanism of the WOX gene family in *N. nucifera*. The proteins interacting with WOX gene family were related to the control of organ identity during the early development of flower, floral meristem development, plant hormone signaling, jasmonate responses, light stress, and regulation of root cell fate. This indicates that the WOX gene family might participate in the network of growth and development, and metabolic regulation in *N. nucifera.*

### 2.9. Subcellular Localization of NnWOX14

**Figure 9:**
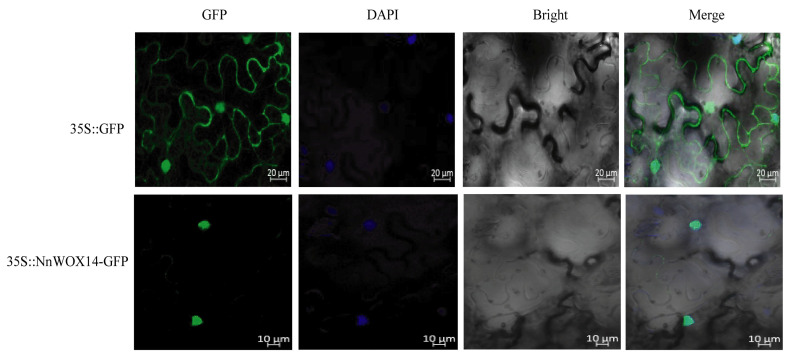
Subcellular localization of *NnWOX14* in tobacco leaf. 35S::GFP was used as the empty control. GFP, green fluorescence protein; DAPI, nuclear marker fluorescence; Bright, bright-field fluorescence; The merged pictures include the green fluorescence channel, nuclear marker fluorescence channel, and bright field fluorescence channel.

Nuclear localizations of transcription factors play an important role in regulating the transcription of target genes by binding to specific cis-elements in their promoters (Leng et al., 2019). In this study, all *NnWOX* proteins were predicted to target the nucleus by CELLO v2.5 (Table 1). To identify the subcellular localization of *NnWOX* proteins, we cloned the *NnWOX14*, which was highly expressed in flower bud and flower, to lay the foundation for follow-up functional verification and interaction research. This gene introduced it into the pMDC202 vector via the CaMV-35S promoter. Then, we transiently co-expressed the *NnWOX14*-GFP fusion protein in *Nicotiana benthamiana* leaves. The green fluorescence signals were observed in the nucleus (Figure 9), suggesting that *NnWOX14* is a nuclear protein. This result is consistent with the predicted result (Table 1).

## 3. Discussion

The WOX TFs play significant roles in the regulation of plant growth and development and response to abiotic stress [2,3,5]. The WOX family members have been identified in model crops, such as *A. thaliana*, rice, maize and poplar [6,10]. However, the WOX gene family has not been identified in *N. nucifera*. In this study, we performed a comprehensive identification of WOX genes in *N. nucifera*. A total of 15 WOX genes were identified in the *N. nucifera* genome. This number is the same as that in *A. thaliana*, while it is higher than the number of WOX genes in rice (13), poplar (12) and sorghum (11), but lower than in maize (21) [6,10]. The homeodomain (HD) of 15 NnWOX proteins was highly conserved and exhibited a typical helix–ring–helix–corner–helix structure (Figure 2), which was consistent with the results in other species [6,10]. This indicates that the homeodomain has been highly conserved throughout evolution. NnWOX proteins were all hydrophilic and unstable, and their similar physical and chemical properties indicated their functional similarity. The subcellular localization of *NnWOX* genes showed their location in the nucleus, which was consistent with the subcellular localization of *OsWOX3*, *OsWOX9* and *OsWOX11* in *O. sativa* and *AtWUS*, *AtWOX3*, *AtWOX4*, *AtWOX6* in *A. thaliana* [3,9,10].

The ML tree of WOXs from five plants showed that they could be divided into three clades and nine subclades (Figure 1), which was consistent with *A. thaliana*, rice, maize, and poplar WOXs [6,9,10]. Gene duplication is the main driving force behind the expansion of gene families in plant genomes [32,33]. We also found that some gene duplication events occurred in the subclades of WOX genes. For example, subclade E, which had two *AtWOX* TFs, two *NnWOX* TFs, one *NcWOX* TF, one *PeWOX* TF, and one *OsWOX* TF, is supposed to undergo one duplication even in eudicots. We also found the gene duplication event in the subclade F, with one duplication event in monocots and eudicots. In addition, five pairs of segmental duplications were observed in *NnWOX* TFs (Figure 5). We, therefore, speculate that gene duplication is associated with the expansion of the WOX gene family, but this needs more research in the future.

In this study, the collinearity map of the *NnWOX* gene family was constructed with ANA grade (*Ny. colorata*), monocots (*O. sativa*), and dicots (*A. thaliana*). There were 13 collinear gene pairs between *N. nucifera* and *A. thaliana*, eight pairs with *O. sativa*, and eight pairs between *N. nucifera* and *Ny. colorata* (Figure 6). The result showed that sacred lotus and *A. thaliana* had a strong linear homologous relationship. At least one member of each clade of *N. nucifera* WOX gene family had a direct homologous relationship with the corresponding member of the *A. thaliana* WOX gene family. In addition, the collinear gene pairs between *N. nucifera* and dicots were more than those between *N. nucifera* and monocots, suggesting that these gene pairs were formed after the differentiation of monocots and dicots. Furthermore, the collinearity analysis also found that four NnWOXs proteins (*NnWOX4*, *NnWOX6*, *NnWOX9*, and *NnWOX13*) and the three selected *WOX* proteins had one or two collinearity gene pairs, which means that these four NnWOXs may exist before ancestral differentiation.

The results of gene structure analysis suggested high structural similarity within each subclade, while there were significant differences among different subclades. This implies that the evolutionary direction of *NnWOX* genes was diverse and conservative in the same subclade, which is in line with the WOX genes of *A. thaliana*, rice, maize, and poplar [6,10,17]. The motif analysis showed that all *NnWOXs* contained tandem Motif 1 and Motif 2, indicating that these genes were conserved among the *NnWOX* family. Our result showed that only the members of the WUS clad contained WUS-box motif (motif 4), which is similar to *A. thaliana* and rice [6,10].

The WOX gene family plays an important role in the regulation of plant growth and development, stress resistance and plant hormone signal transduction [4,6]. The promoter of *NnWOXs* contains cis-acting elements related to plant hormones and stress. Auxin can regulate embryonic development, abscisic acid affects seed dormancy and germination, and methyl jasmonate (MeJA) is involved in plant defence response [12,26,27]. The WOX gene is regulated by hormones such as IAA, ABA and GA in the regulation of plant growth and development [6]. Many hormone response elements were observed in the *NnWOX* promoter region, including cis-acting elements ABRE, auxin responsiveness element, TGA-element, TCA-element and GARE-motif involved in ABA, IAA, SA and GA responses, respectively. Therefore, the *NnWOX* gene family plays a pivotal role in plant growth, and development and resistance to stress by mediating plant hormone regulation in *N. nucifera*.

In this study, we analyzed the expression patterns of 15 *NnWOX* genes in the root, leaf, flower, rhizome, petiole, petal, receptacle, carpel, stamen, seed coat, and cotyledon of *N. nucifera*. *NnWOX14* and *NnWOX15* were highly expressed in most organs, and showed a close relationship with *AtWOX13* in the evolutionary tree. *AtWOX13* is expressed during the development of primary root and lateral root of *A. thaliana*. It is also highly expressed in the inflorescence and flower bud, showing its involvement in the development of lateral root and the formation of flower organs [11]. Therefore, *NnWOX14* and *NnWOX15* may involve root growth and formation of flower organs. The expression of WUS gene in the central region of shoot apical meristem is necessary for the formation and maintenance of shoot apical meristem, and WUS gene is also involved in the process of ovary and anther development [14,25]. *NnWOX3* is homologous to WUS and it showed little or no expression in different organs, which is maybe due to difference in sampling stages. *OsWOX11* is specifically transcribed in root meristem and stimulates crown root formation and growth in rice, which regulates crown root development via the cytokinin and auxin pathway [34,35]. The *NnWOX10* was clustered in the same subclade with *OsWOX11* (Figure 1). The cis-element analysis showed that *NnWOX10* contained two auxin responsiveness elements, indicating that this gene might be related to auxin metabolism. However, *NnWOX10* showed little expression in root, which might be caused by the difference in the sampling stage. Duplication and polyploidy events could increase the family genes in plant species [32,33]. According to our results, the duplicated WOX genes have acquired new functions. It seems that some mutations in coding sequence site as well as promoter region have caused the diversity of expression patterns and new genes have received the diverse functions [36]. GO annotations showed that the *N. nucifera* WOX gene family was involved in the development of embryos and other tissues and organs, and intercellular communication as nuclear-localized transcription factors.

## 4. Materials and Methods

### 4.1. Identification of WOX Gene and Physicochemical Properties Analysis

We downloaded the *N. nucifera* genome data from the Nelumbo Genome Database (http://nelumbo.biocloud.net, accessed on 1 April 2022) [37]. We downloaded the genome data of *A. thaliana*, *O. sativa*, and *Ny. colorata* from the National Center for Biotechnology Information (NCBI, https://www.ncbi.nlm.nih.gov/, accessed on 1 April 2022). The WOX protein sequences of *A. thaliana*, *Phalaenopsis equestris*, and *O. sativa* were used as query sequences. A local BLASTp search was conducted using *A. thaliana* and *O. sativa* WOX protein sequences as the query using Tbtools (e-value ≤ e^−10^) [38]. Then, the blast results were searched on the NCBI website. The NCBI Batch CD-search was used to analyze the structure of candidate WOX proteins, and the members without typical conserved domains of WOX proteins were removed. All members of the WOX transcription factor family were obtained. The protein sequences of the WOX family were analyzed on the ExPasy website (http://au.expasy.org/tool.html, accessed on 22 April 2022) using the ComputepI/MW function to ascertain the physicochemical properties [39]. Finally, the CELLO v2.5 website (http://cello.life.nctu.edu.tw/, accessed on 22 April 2022) was used to predict the subcellular localization of *N. nucifera* WOX proteins.

### 4.2. Construction of Phylogenetic Tree

The ClustalW function of Mega 11 was used to make multiple alignments of WOX protein sequences (Supplementary Data S1) [40]. Phylogenetic analysis was performed using the maximum-likelihood (ML) method. The ML tree was constructed on the CIPRES Science Gateway website (https://www.phylo.org/, accessed on 23 April 2022) [41] with the following settings: sampling frequency = 1000; tem = 0.1; burn-in = 2000.

### 4.3. Conservative Motif, Gene Structure, and Cis-Acting Elements Analysis

We used the ClustalW function of Mega 11 to align the NnWOX protein sequences, visualized using GeneDoc [42]. The conserved motifs of the WOX gene in *N. nucifera* were searched on the MEME website (http://meme-suite.org/tools/meme, accessed on 17 May 2022) [43], and visualized by Tbtools [38]. The identification of cis-acting elements in the upstream 2 kb promoter region of the *N. nucifera* WOX gene family was performed on the PlantCARE website (http://bioinformatics.psb.ugent.be/webtools/plantcare/html/, accessed on 20 May 2022) [44], and Tbtools was used to extract the upstream 2 kb sequences. Finally, Microsoft Excel 2010 was used to visualize the results. Tbtools was used to analyze the structure of *N. nucifera* WOX gene family.

### 4.4. Chromosome Location and Collinearity Analysis of NnWOXs

According to the annotation gff3-file of *N. nucifera* [37], the chromosome location information of WOX transcription factor was extracted, and analyzed and visualized by Tbtools [38]. The one-step MCScanX function of TBtools was used to analyze the intraspecific and interspecific collinear relationships of the WOX gene family [38].

### 4.5. Gene Expression Pattern Analysis, GO Annotation, and Interaction Protein Analysis

We downloaded the gene expression matrix of different organs and Gene Ontology annotations of ‘China Antique’ from the Nelumbo Genome Database (http://nelumbo.biocloud.net, accessed on 1 April 2022), visualized by TBtools [38] and Excel 2010, respectively. The organs such as root, leaf, flower bud, rhizome, petiole, petal, receptacle, carpel, stamen, seed coat, and cotyledon were used in this study. The gene network analysis was predicted by the STRING website (https://cn. string-db.org/, accessed on 12 June 2022) and visualized by Cytoscape [45,46].

### 4.6. RNA Extraction and qPCR Analysis

The root, rhizome, leaf, flower bud, and booming flower were sampled from *N. nucifera* planted at the Fujian Agriculture and Forestry University. The root, rhizome, and leaf were sampled at blooming period. The FastPure ^®^ Plant Total RNA Isolation Kit (Polysaccharides and Polyphenolics-rich) (Vazyme, Nanjing, China) was used to extract the total RNA. The Nanodrop 2000 spectrophotometer was used to determine the concentration of total RNA, and the integrity of total RNA was detected by agarose gel electrophoresis. The HiScript III 1st Strand cDNA Synthesis Kit (+gDNA wiper) (Vazyme, Nanjing, China) was used to reverse transcribe RNA into cDNA. The Primer3Plus online tool was used to design the qRT-PCR primers (Supplementary Table S1), and the actin gene was used as a reference. The qRT-PCR was performed by using Taq Pro Universal SYBR qPCR Master Mix kit (Vazyme, Nanjing, China). Finally, the 2^−∆Ct^ method was used to calculate the expression level [1].

### 4.7. Cloning and Subcellular Localization Analysis

Snapgene 3.2.1 was used to design the primers of *NnWOX14*, removed the stop codon and, added *Xba*I and *Kpn*I restriction sites to the 5′ end, respectively. The following primers were used: forwards: GGACCTCGACTCTAGAATGGGTACGGTAAGAAATGCTG, reverse: TCATTTTTTCTACCGGTACCTTGTTCTTTGGAATGAAAGTTATGC. The PCR amplification was performed and the product was purified. Then, the fragment was ligated with the pMDC202 vector (35S::GFP) [1] and transformed into *Escherichia coli* (DH5α). The recombinant plasmid was extracted and transferred to *Agrobacterium tumefaciens* (GV3101) using the freeze–thaw method. Finally, the product was transformed into tobacco leaf, and the localization of *NnWOX14* was observed under laser confocal microscope (ZEISS, LSM 880) after 48 h of culture with no-load pMDC202-35S-GFP as control, and the nucleus-specific dye (DAPI) was used as nuclei dye.

## Figures and Tables

**Table 1:** Characteristics of the WOX gene family in *N. nucifera*.

Gene	Number of Amino Acids	Molecular Weight (Average)	Theoretical PI	Instability Index	Aliphatic Index	Grand Average of Hydropathicity (GRAVY)	Subcellular Localization
*NnWOX1*	328	35,913.4	6.09	52.55	71.65	−0.377	Nuclear
*NnWOX2*	216	24,472.7	9.46	55.66	64.07	−0.935	Nuclear
*NnWOX3*	273	30,026.25	7.59	67.62	51.47	−0.794	Nuclear
*NnWOX4*	366	40,294.14	8.22	56.67	65	−0.547	Nuclear
*NnWOX5*	190	21,804.68	8.62	69.09	61.68	−0.737	Nuclear
*NnWOX6*	182	20,640.22	7.72	59.93	67.47	−0.778	Nuclear
*NnWOX7*	253	27,897.2	6.76	55.71	60.91	−0.63	Nuclear
*NnWOX8*	200	23,237.49	9.07	67.16	57.15	−0.802	Nuclear
*NnWOX9*	185	20,976.68	8.7	42.02	70.05	−0.63	Nuclear
*NnWOX10*	281	30,518.08	5.42	74.84	69.68	−0.289	Nuclear
*NnWOX11*	338	38,499.82	5.77	52.98	57.49	−0.806	Nuclear
*NnWOX12*	345	39,330.66	6.06	57.13	53.45	−0.905	Nuclear
*NnWOX13*	214	24,352.59	9.46	55.43	65.65	−0.916	Nuclear
*NnWOX14*	268	30,902.74	6.08	59.61	64.78	−0.87	Nuclear
*NnWOX15*	280	31,838.8	5.65	58.63	68.29	−0.758	Nuclear

**Table 2:** The Ka/Ks of *NnWOXs*.

Gene 1	Gene 2	Ka	Ks	Ka/Ks
*NnWOX2*	*NnWOX13*	0.090611	0.4671997	0.193945
*NnWOX6*	*NnWOX9*	0.0633006	0.5448065	0.1161891
*NnWOX5*	*NnWOX8*	0.0991806	0.7733448	0.1282488
*NnWOX11*	*NnWOX12*	0.0896248	0.3670029	0.2442074
*NnWOX14*	*NnWOX7*	0.1293221	0.4279474	0.3021915

## Data Availability

Not applicable.

## Supplementary Materials

The following supporting information can be downloaded at https://www.mdpi.com/article/10.3390/ijms241814216/s1.
