# Supplementary material for: Genome-Wide Analysis of WUSCHEL-Related Homeobox Gene Family in Sacred Lotus (Nelumbo nucifera)
Source: Int J Mol Sci. 2023 Sep 18;24(18):14216. doi: 10.3390/ijms241814216 (PMC10531982; doi:10.3390/ijms241814216)
Supplement: Supplementary file 1 [file ijms-24-14216-s001.zip › ijms-2559298-SI.pdf]

Supplementary Material

Supplementary Data 1 WOX protein sequences of *N. nucifera*, *A. thaliana*, *O. sativa*, *Nc. Colorata*, and *P. equestris*.

>PEQU_01751
MNDFHGRLYEEERAMGDLQGMLDGVLFMKVMTDEQIEVLRRQISIYATICEQLVAMHRAF
TKTLAGNNFGTTSYNPSASAEVPKIAARQRWIPTSTQLQILENIFKQGYETPSKQKIAEITSKL
AKHGHVSDVKVYNWFQNKRARSKKKQAVLLKSNAEQENEAEIESCSRKKMKMDENHENE
EPELNRGEEEHASMDSAGVLCAQEQVNFYENSASLPSKWSHSFSSV
>PEQU_10674
MDNNQPQEPTTNTVIPSSGELPASRTNAVSDPIRSRWTPKPEQILILESIFNSGMVNPPREETV
RIRKLLEKFGSVGDANVFYWFQNRRSRSRRRQRQLQASLAAATSTYESTAASCSSSSLDAAS
LTSSFGSSPSSSSPPSSMGSVWCESADDLFSISQQMGFGDSTSSSNRICCRINTEITIQNYNAGT
VTVFINEILTEVPRGPIDIRSMFGHDVLIVHSSGEFLPVNEYGILLQSLQMGESYYLVIFIAKIAF
Y
>PEQU_10733
MPQVPSTRWCPTPEQLMILEEMYRSGVRTPNASQIQQITTHLSYYGKIEGKNVFYWFQNHK
ARERQKLRRRLTKQQQQQQHPIQFQNLEDASLPLHDDSSQMLYGQQMNPQFFQQVALSPVS
SCAFSFYLIELASLHVRRYEVHATKLGFLLFEEVLSL
>PEQU_12866
YVKVMTDEQMEILRRQIAVYATICEQLVEMHKAVSAQQDSLAGLKYNCSFGNVVMRLLLY
LFGPGMRFGSLYCDPLMTSTTHKLASRQRWTPTPMQLQILETIFDQGNGTPTKQKIKEVTLE
LTKHGQISETNVYNWFQNRRARSKRKQSSAATSITESEADTESESRNDKRAKAETALSEDLP
NPPPSLQ
>PEQU_12926
MDLFSAGLRTPSTEQIQKISYHLSSFGKIENKNVFYWFQNHKARERHHHPQKKRRRPPPAPTS
PVHSELFVIEPSEHRKFPVTKLSGLSSYHSKDVKYLRRILL
>PEQU_16293
MEGNQNSSGNIPPAASEPIRSRWTPKPEQILILESIFESGMVNPAKDETVRIRKLLEKFGSVGD
ANVFYWFQNRRSRSRRRQRQIQASLAAASAVVGGGKTTGFEQSAAVAAAMASCSSNCTSSS
SSSSSSGGGGGAGFYYGASSSVTPSSVSLLSGFGGSHKADDLFSISDQMGGLGDDSYMSCYE
AAQMQYQTGSITVFINGIPSEVPSGLINIRAMFGHDAMLIHSSGEPLPINDCGILLQSLQMGES
YYLVRLKIRCSLSIYISSNIARSHSLK
>PEQU_18124
MRTPNAQQIEKITAELMNHGRIEGKNVFYWFQNHKARERQKQKRNAHLSPSSPICSDTKA
>PEQU_28508
MEWVKVPTSFNDGEDRRRGVLYVKVMTDEQMEILRCQIAVYAIICEQLVKMHRAVSAQLD
SLAGMRFGSLYCDPVMTSTTHKLASKLNTNTHVALILESIFDQGNGTSSKQKIKEVIFELTKR
VKISETNVYNWFQNRRARAKRKQSSVATSITESEADTQSESRNDKKAKTE
>PEQU_33785
CLFMEPNCHSQPQEENGNPGKSSSFLCRQSSARWIPTGDQIRILRDLYYNYGVRSPSAEQIQRI
SAKLRQYGKIEGKNVFYWFQNHKARERQKKRLTSDIASSNNSNVINSKYFVHTIVLFLHT
>PEQU_35566
YVKVMTDEQMEILRRQIAVYATICEQLVEMHKAVSAQQDSLAGLKYNCSFGNVVMRLLLY
LFGPGMRFGSLYCDPLMTSTTHKLASRQRWTPTPMQLQILETIFDQGNGTPTKQKIKEVTLE
LTKHGQISETNVYNWFQNRRARSKRKQSSAATSITESEADTESESRNDKRAKAETALSEDLP
NPPPSLQ
>PEQU_38029

MEEEKPPSPPSSSSSLPVPSPSTRWNPTKEQITILEGLYWQGIRTPTADQIQHITSKLREFGSIEG
KNVFYWFQNHKARQRQKQKQESFAYFSRLLHRHPSTQPPTPPPPPPGICCSNGLFIFFPLMCY
IFTNRHFLNVHCYICAN
>PEQU_40270
MPQVPSTRWCPTPEQLMILEEMYRSGVRTPNASQIQQITSHLSFYGKIEGKNVFYWFQNHKA
RDRQKLRRRLSRQQAQVSSSSLPPHPPPQFLPHLYPEFNSQFFQQV
>PEQU_41149
MVNPPRDEIRRIRSQLQVYGQVGDANVFYWFQNRKSRSKHKQRHLKSTPAAAAAAKSTPPP
LTPASSTSTSGSNTKQIVTTLPALSTFFDQNFLQAPILMPELSLESSVLLPASYQISSGELSGGSP
AAWDLIMQEVFPSKSAGASIGCVVSGDEEIAVATGTATPGTVNEIKGTLYIILK
>AT1G20700.1
MVKKKKEKEKSKEIEEMDREIQNGAYSGRVMTEEQMEILRKQIAVYAVICDQLVLLHNSLS
SYHPLSSGVRPMVGGYFDPMGASSSSHRISTRHRWTPTSTQLQILESIYDEGSGTPNRRRIREI
ATELSEHGQITETNVYNWFQNRRARSKRKQPQTTTANGQADDVAVTTEERRSCGDSGGLES
YEHILFPSPDLGIEHLLSIGKFMET
>AT1G20710.1
MEQESLNGRYGSRVMTDEQMETLRKQIAIYAVLCDQLVFLHNSLSSVPLLSSGMNPMRGEY
FDPMVASSSAHGMSTRPRWTPTTTQLQILENIYKEGSGTPNPRRIKEITMELSEHGQIMEKNV
YHWFQNRRARSKRKQPPTTTITSSQADDAAVTTTEERGRCGDDSGGFESYEHILFPSPDLGIE
HLLNRDKFID
>AT1G46480.1
MKVHEFSNGFSSSWDQHDSTSSLSLSCKRLRPLAPKLSGSPPSPPSSSSGVTSATFDLKNFIRP
DQTGPTKFEHKRDPPHQLETHPGGTRWNPTQEQIGILEMLYKGGMRTPNAQQIEHITLQLGK
YGKIEGKNVFYWFQNHKARERQKQKRNNLISLSCQSSFTTTGVFNPSVTMKTRTSSSLDIMR
EPMVEKEELVEENEYKRTCRSWGFENLEIENRRNKNSSTMATTFNKIIDNVTLELFPLHPEGR
>AT2G01500.1
MGYISNNNLINYLPLSTTQPPLLLTHCDINGNDHHQLITASSGEHDIDERKNNIPAAATLRWN
PTPEQITTLEELYRSGTRTPTTEQIQQIASKLRKYGRIEGKNVFYWFQNHKARERLKRRRREG
GAIIKPHKDVKDSSSGGHRVDQTKLCPSFPHTNRPQPQHELDPASYNKDNNANNEDHGTTEE
SDQRASEVGKYATWRNLVTWSITQQPEEINIDENVNGEEEETRDNRTLNLFPVREYQEKTGR
LIEKTKACNYCYYYEFMPLKN
>AT2G17950.1
MEPPQHQHHHHQADQESGNNNNNKSGSGGYTCRQTSTRWTPTTEQIKILKELYYNNAIRSP
TADQIQKITARLRQFGKIEGKNVFYWFQNHKARERQKKRFNGTNMTTPSSSPNSVMMAAN
DHYHPLLHHHHGVPMQRPANSVNVKLNQDHHLYHHNKPYPSFNNGNLNHASSGTECGVV
NASNGYMSSHVYGSMEQDCSMNYNNVGGGWANMDHHYSSAPYNFFDRAKPLFGLEGHQ
EEEECGGDAYLEHRRTLPLFPMHGEDHINGGSGAIWKYGQSEVRPCASLELRLN
>AT2G28610.1
MSPVASTRWCPTPEQLMILEEMYRSGIRTPNAVQIQQITAHLAFYGRIEGKNVFYWFQNHKA
RDRQKLRKKLAKQLHQQQHQLQLQLQQIKPKPISSMISQPVNKNIIDHHNPYHHHHHNHHH
NHHRPYDHMSFDCCSHPSPMCLPHQGTGVGEAPSKVMNEYYCTKSGAEEILMQKSITGPNS
SYGRDWMMMMDMGPRPSYPSSSSSPISCCNMMMSSPKIPLKTLELFPISSINSKQDSTKL
>AT2G33880.1
MASSNRHWPSMFKSKPHPHQWQHDINSPLLPSASHRSSPFSSGCEVERSPEPKPRWNPKPEQI
RILEAIFNSGMVNPPREEIRRIRAQLQEYGQVGDANVFYWFQNRKSRSKHKLRLLHNHSKHS
LPQTQPQPQPQPSASSSSSSSSSSSSKSTKPRKSKNKNNTNLSLGGSQMMGMFPPEPAFLFPVST
VGGFEGITVSSQLGFLSGDMIEQQKPAPTCTGLLLSEIMNGSVSYGTHHQQHLSEKEVEEMR

MKMLQQPQTQICYATTNHQIASYNNNNNNNNNIMLHIPPTTSTATTITTSHSLATVPSTSDQLQ
VQADARIRVFINEMELEVSSGPFNVRDAFGEEVVLINSAGQPIVTDEYGVALHPLQHGASYY
LI
>AT3G03660.2
MDQEQTPHSPTRHSRSPPSSASGSTSAEPVRSRWSPKPEQILILESIFHSGMVNPPKEETVRIRK
MLEKFGAVGDANVFYWFQNRRSRSRRRQRQLQAAAAAADATTNTCDQTMMVSNSLPHHS
GSDLGFGGCSTSSNYLFGGSSQVPSFFLGLSSSPSSCSSSSSTSSSASSSSSYGGGCDNQSNSG
MENLLTMSGQMSYHEATHHHYQNHSSNVTSILCPSDQNSNFQYQQGAITVFINGVPTEVTR
GGIDMKATFGEDLVLVHSSGVPLPTDEFGFLMHSLQHGEAYFLVPRQT
>AT3G11260.1
MSFSVKGRSLRGNNNGGTGTKCGRWNPTVEQLKILTDLFRAGLRTPTTDQIQKISTELSFYG
KIESKNVFYWFQNHKARERQKRRKISIDFDHHHHQPSTRDVFEISEEDCQEEEKVIETLQLFP
VNSFEDSNSKVDKMRARGNNQYREYIRETTTTSFSPYSSCGAEMEHPPPLDLRLSFL
>AT3G18010.1
MWTMGYNEGGADSFNGGRKLRPLIPRLTSCPTAAVNTNSDHRFNMAVVTMTAEQNKREL
MMLNSEPQHPPVMVSSRWNPTPDQLRVLEELYRQGTRTPSADHIQQITAQLRRYGKIEGKN
VFYWFQNHKARERQKRRRQMETGHEETVLSTASLVSNHGFDKKDPPGYKVEQVKNWICSV
GCDTQPEKPSRDYHLEEPANIRVEHNARCGGDERRSFLGINTTWQMMQLPPSFYSSSHHHH
QRNLILNSPTVSSNMSNSNNAVSASKDTVTVSPVFLRTREATNTETCHRNGDDNKDQEQHE
DCSNGELDHQEQTLELFPLRKEGFCSDGEKDKNISGIHCFYEFLPLKN
>AT4G35550.1
MMEWDNQLQPNNHHSSNLQGIDVNGGSGAGGGMYVKVMTDEQYETLRKQIAIYGTICERL
VEMHKTLTAQQDLAGGRMGGLYADPMMSSLGHKMTARQRWTPTPVQLQILERIFDQGTGT
PSKQKIKDITEELSQHGQIAEQNVYNWFQNRRARSKRKQHGGGSSGNNNGESEVETEVEAL
NEKRVVRPESLLGLPDGNSNNNGLGTTTATTTAPRPEDLCFQSPEISSDLHLLDVLSNPRDEH
LVGKMGLAESYNLYDHVEDYGMSG
>AT5G05770.1
MSSRGFNIKARGLCNNNNGGGGTGAKCGRWNPTVEQVKLLTDLFKAGLRTPSTDQIQKISM
ELSFYGKIESKNVFYWFQNHKARERQKCRKISTVKFDHRQDTDLSKPRRDNVRRHQLPAKG
>AT5G17810.1
MNQEGASHSPSSTSTEPVRARWSPKPEQILILESIFNSGTVNPPKDETVRIRKMLEKFGAVGD
ANVFYWFQNRRSRSRRRHRQLLAATTAAATSIGAEDHQHMTAMSMHQYPCSNNEIDLGFG
SCSNLSANYFLNGSSSSQIPSFFLGLSSSGGCENNNGMENLFKMYGHESDHNHQQQHHSSN
AASVLNPSDQNSNSQYEQEGFMTVFINGVPMEVTKGAIDMKTMFGDDSVLLHSSGLPLPTD
EFGFLMHSLQHGQTYFLVPRQT
>AT5G45980.1
MSSSNKNWPSMFKSKPCNNNHHHQHEIDTPSYMHYSNCNLSSSFSSDRIPDPKPRWNPKPEQ
IRILESIFNSGTINPPREEIQRIRIRLQEYGQIGDANVFYWFQNRKSRAKHKLRVHHKSPKMSK
KDKTVIPSTDADHCFGFVNQETGLYPVQNNELVVTEPAGFLFPVHNDPSAAQSAFGFGDFVV
PVVTEEGMAFSTVNNGVNLETNENFDKIPAINLYGGDGNGGGNCFPPLTVPLTINQSQEKRD
VGLSGGEDVGDNVYPVRMTVFINEMPIEVVSGLFNVKAAFGNDAVLINSFGQPILTDEFGVT
YQPLQNGAIYYLI
>AT5G59340.1
MENEVNAGTASSSRWNPTKDQITLLENLYKEGIRTPSADQIQQITGRLRAYGHIEGKNVFYW
FQNHKARQRQKQKQERMAYFNRLLHKTSRFFYPPPCSNVGCVSPYYLQQASDHHMNQHGS
VYTNDLLHRNNVMIPSGGYEKRTVTQHQKQLSDIRTTAATRMPISPSSLRFDRFALRDNCYA
GEDINVNSSGRKTLPLFPLQPLNASNADGMGSSSFALGSDSPVDCSSDGAGREQPFIDFFSGG
STSTRFDSNGNGL

>Nn2g12813.1
MSPAASTRWCPTPEQIMILEEMYRGGIRTPNATQIQQITAHLSFYGKIEGKNVFYWFQNHKA
RERQKLRRKLCRQQQQQLSAHHHRFLHQIEQSDSSALHQPPLYNSAQLLPQAGGAKEASND
VMSYSSRKMDIPERDEKERSKWMYGRDWMMMMMMDIGPTTHCCNRPLKTLQLFPITTTSL
KDECTTSNHLSCSTSTD
>Nn2g10575.1
MSPAASTRWCPTPEQLMILEEMYRGGIRTPNATQIQQITAHLSFYGKIEGKNVFYWFQNHKA
RERQKLRRKLSRQQQQQQQQYPHHHFLHQIDPSDAAAAALQQIPLYNSTQIVPQIGGLKES
SSNDDIERSTRMYGRDWMMMMMDVGPATPCCNRPLKTLQLFPITTSSSLKDECTTSDHLSC
STSSD
>Nn5g30363.1
MRSLNMKVHQLARGLWEHEPSLTLGCKRLRPLAPKLTNGETPAILDLKSFIKPESSSGPSKSD
DKREAVQVDAPSGGTRWNPTQEQIGILEMLYRGGMRTPNAQQIEKITAQLGKFGKIEGKNV
FYWFQNHKARERQKQKRNSLGLVHSPRTPPATTVALDVNRDEIEREEDSHYKRKHRGWAIE
FFQQDRGFCGGGEEGDKTLELFPLHPEGR
>Nn2g12791.1
MEDKSVELLASAGSPASTRWNPTKEQISILESLYRQGIRTPTAEQIQQISSRLRMYGHIEGKNV
FYWFQNHKARQRQKQKQESMVYANQFFHKAAPVLVHSPRPCNNVVCAPFYMPQTGIGFYP
PHPKVLLPGGVKRKPKMDKMEKRKGTGGGGDGVSAGAGYEAAQAGFYNMYKDDDNEGL
VGTTDGHHETLTLFPLHPTGVLEDKIENHFPSTSAENSNASTSSGSNSVEVVSADQPLIDFFAE
NGSVESY
>Nn1g04777.1
MGSLNMKVHQLARGLWEHEPSLTLGCKRLRPLVPKLTNGDSVSNLDLKSFIKPESSPKKPAG
STDEKRQPVQVEAPSGGTRWNPTQEQIGILEMLYRGGMRTPNAHQIEQITAQLGKFGKIEGK
NVFYWFQNHKARERQKQKRNSLGLTHSPRIAPPTTTVTLDPGREVEREEDSHYKRKHRSWG
VEFFGEDSSRLCGGEEGDKTLELFPLHPEGR
>Nn3g18264.1
MWMMGCSDGSGFNMPDSFNSRKLRPLMPRLTTNSTNSAAGIAPCLSRIHGTDLFALNTHLA
TVNEQSKREFISAQPVVSSRWNPTPEQLRTLEELYRCGTRTPTAEQIQHITAQLRRFGKIEGK
NVFYWFQNHKARERQKRRRRAAEEQQHCDTENLDRKESGSSKTGFGIEQTRNWTWAPPTL
AEETVSMQRAAVAESRTDGWIQFEEGELQQRSSSVERHATWQMMQLSSSCPSTTLISNITTA
TTEIDPKLVNNRPLDIFKTTCSTEHLSTLLINGEDRNGEEECGESQTLELFPLQSERNNGSDDA
EKETEVANPTMNTNFTSYQFFEFLPMKN
>Nn4g24211.1
MWMMGCSDGSGFNMADSFNGRKLRPLMPRLTTNSTNTATVMAPCLTRIHGTDFFALNTHL
ATVTEHSKREFSAQPVVSSRWNPTPEQLRTLEELYRCGTRTPTAEQIQHITAQLRRFGKIEGK
NVFYWFQNHKARERQKRRRRSAASEEQQHYDTESLDKKESGSSRTGYEVEQTKNWAPPTN
CSSLSEETVSMQRAAVAESRTDGWIQFEEGELQHRRTSVERNPTWQMMQLSSPPTTPLKNTI
TTATTEAITVVDPKLINNQNLELFKTPSREYLPADLLSNGEERKDQEECGESQTLQLFPLRSDS
GNGGVGDEEKETEVPNPTTNSNFTTYQFFEFLPTKN-
>Nn2g11407.1
MDERMSGFCLRAGGGGYGGGDHGGGGGGTKCGRWNPTAEQVKVLTDLFRSGLRTPSTDQI
QKISAQLSFYGKIESKNVFYWFQNHKARERQKRKRVSVEDRDIIERAADKVLPEKHFVDVDL
VSEPERTIETLQLFPLNSYEESEPQKLRLLRNECKESAFSYNGMGKENDHPPLDLRLSFL
>Nn1g06358.1
MASSNRHWPSMFKSKPCNTHHQWQHDINPSLMSTPCQRTPYAPVPGCEERSPEPKPRWNPK
PEQIRILEAIFNSGMVNPPRDEIRKIRAQLQEYGQVGDANVFYWFQNRKSRSKHKQRHLQSA

KSQAQTAPVTTSPAPAPAVAVQASSSSSSSEKSPPRRTDKPLPAGGDNVIEASNSPTTSVNQT
YFQPQLHVDFNPEPFFFPVQSPSFSQGFCFSELSNVVQIPDQLTNPCPDSLLLNDLINQGAPKK
DDQVKMKLNQQLSSYTTITTAPVTTTTTGYTTALNQIQGVGESGGNGMIPAAAVKSTVFIND
VAFEVAVGPLNVREAFGDDAILVNSSGQPVLTNEWGVTLQSLQHGAFYYLWRTV
>Nn2g13641.1
MDERMLGFCIRAGGYGYGGGGCHAAGGGGGGGTKCGRWNPTAEQVKVLTDLFKSGLRTPS
TDQIQKISTQLSFYGKIESKNVFYWFQNHKARERQKRKRVSVDDQDFIDRAVDKVLPATKHF
VEVDLVSEPERTIETLQLFPLNSYQDSDSLKLRLFRNEYKETTFSYGIGKENDHPPLDLRLSFL
>Nn1g04282.1
MNVYSVAVTGCEERSPEPKPRWNPKPEQIRILEAIFNSGMVNPPRDEIKRIRAQLQEYGQVG
DANVFYWFQNRKSRSKHKQRHLQSAKAQTLQTIPITTAPATTAVLPSSSSSSSEKSPPRRTDK
TPPMGGTNMIEASNSPTASVNQTYFHPQVDFQPEPFLFPVQPPPSFSQEFCFSELSNVVQASDS
DHQLTWPCSGGLLLNDIINQGASKKDDQVKMKPTQQFNYSTISTAPVTSAIASTTTATLNQIQ
GVDESGGMIPAAVAKSTVFINDVAFEVAMGPLNVREAFGEDAVLVHSSGLPVITDEWGVTL
QALQHGAFYYLVRAV-
>Nn1g05878.1
MEPQQQPAQQMQQQQQPNEDSSSSNTSKSSFLCRQSSTRWTPTTDQIRILKDLYYNNGIRSPS
AEQIQRISARLRQYGKIEGKNVFYWFQNHKARERQKKRFTVDMAMQRSVATGGWKTGDPI
HNKYTNISPGVSSPSSPGVLAAGQMGGTGYGPVLMEKSFRDCSISAGGSGIGGGGMCNNLG
WVGIDPYSSPYALFDKRKSIEYLEDQTPQIETLPLFPTHGENIGGFCTGKADSGGYFTDWYGP
SDEKNDTPTSLELSLNSCTTTSPDSP
>Nn2g14576.1
MIACVCSPHLSDSGFWYFPTSLILSAMEDQVGDANNPSHGSERTEPVRSRWTPKPEQILILESI
FNSGMVNPPKDETVRIRKLLEKFGTVGDANVFYWFQNRRSRSRRRQRQIQAGLAGDPRIPG
RATGAIQYEPTSTSACFAASSSPTFSSFPSSSSSSSSSSSSLVGSSSSSCGGDGVDDLFAISRQM
GVPETEQSPTVDSILCPSDTPNFHYQSGLITVFINGIPTEVPRGPLDLRSIFGEDVMLVHSSGVP
VPFNEYGVSTQSLQMGETYFLVPRQI
>Nn5g30993.1
MGTVRNAVEGEEKVRIMEWEKQEQQQQPQQQQNAGVMYVKVMTDEQMELLRKQISIYA
TICEQLVEMHKAITAQQDLAGMRLGNLYCDPLMASSGHKITARQRWTPTPIQLQILERIFDQ
GNGTPSKQKIKEITSELTQHGQISETNVYNWFQNRRARSKRKQQVSIPNNAESEVETEVESPK
EKKTKPENIHFHENQAQKAEDLCFQSAEISSELHSLDPHPTKMEATFPLDGTSKSSGSLTHMS
FYESVLSNPIMYWHNFHSKEQ
>Nn6g31691.2
MGTIRNGGETKEEMKMMEWEKQEQQQQQNGVMMYVKVMTDEQMELLRKQISIYATICE
ELVEMHKAITAQRDLAGMRLGNLYFDPLMTSSGHKITARQRWTPAPIQLQILERIFDQGNGT
PSKQKIKEITSELAQHGQISETNVYNWFQNRRARSKRKQQVAVPNNAESEIEIEVESPDEKKA
KPEIIHLHENQAPSAEDMCYQSPGIISALHSVDPQHNKAESIFSSDNTSKSSVSLSHLSFYESVL
SNSGYDHLIGKMEGPGSFNPYRQGEGYDMIG
>XP_031473186.1
MASSNRHWPSMFKSKPCNSHHHQWQHDIISACQKQPYSSVGGEERSPEPKPRWNPKPEQIRI
LEAIFNSGMVNPPRDEIRKIRAQLQEYGQVGDANVFYWFQNRKSRSKHKQRHLSGKTHLST
PTSTSMAATPPPQMNTITATATAAATAATTTNTATVTTTTTTTNSSSSSERTSSSAYLNAME
SVNSPSTPTASVNQAGFYQPALHQFPLHDPRSFFFQNSAPFPSLSAPSSLSELSSMVQLHEQLK
QDERLNVVVGSPHQNYAIAPLLQDLGGSASSSARLTVFINDLAFEVAPMPVNVREAFGEEAV
LLHSSGQPVMTDEFGVTLQPLQNGAFYYLV
>LOC_Os01g47710.1

MASSNRHWPSMFRSKHATQPWQTQPDMAGSPPSLLSGSSAGSAGGGGYSLKSSPFSSVGEE
RVPDPKPRWNPRPEQIRILEAIFNSGMVNPPRDEIPRIRMQLQEYGQVGDANVFYWFQNRKS
RSKNKLRSGGTGRAGLGLGGNRASAPAAAHREAVAPSFTPPPPILPAPQPVQPQQQLVSPVA
APTSSSSSSSDRSSGSSKPARATSTQAMSVTTAMDLLSPLAAACHQQMLYQGQPLESPPAPA
PKVHGIVPHDEPVFLQWPQSPCLSAVDLGAAILGGQYMHLPVPAPQPPSSPGAAGMFWGLC
NDVQAPNNTGHKSCAWSAGLGQHWCGSADQLGLGKSSAASIATVSRPEEAHDVDATKHGL
LQYGFGITTPQVHVDVTSSAAGVLPPVPSSPSPPNAAVTVASVAATASLTDFAASAISAGAVA
NNQFQGLADFGLVAGACSGAGAAAAAAAPEAGSSVAAVVCVSVAGAAPPLFYPAAHFNVR
HYGDEAELLRYRGGSRTEPVPVDESGVTVEPLQQGAVYIVVM
>LOC_Os01g60270.1
MEWDKAKASSGEAVDDRGGGEGGLGYVKVMTDEQMEVLRKQISIYATICEQLVEMHRAL
TAQQDSIAGMRLGNLYCDPLMVPGGHKITARQRWTPTPMQLQILENIFDQGNGTPSKQKIK
DITAELSQHGQISETNVYNWFQNRRARSKRKQAALPNNNAESEAEADEESPTDKKPKSDRPL
HQNIAMRDHNSERISEMHHFDTEHEQIRRMMYASNDSSSRSSGSLGQMSFYDNVMSNPRID
HFLGKVESPGSFPHMRSGESFDMY
>LOC_Os01g62310.1
METTTTTLGGGGGGRAGGFSDPPSPLSPPLSPASAAAAALANARWTPTKEQIAVLEGLYRQG
LRTPTAEQIQQITARLREHGHIEGKNVFYWFQNHKARQRQKQKQQSFDYFSKLFRRPPPLPV
LHRPLARPFPLAMAPTAMPPPPPPPATTTTAACNAGGVMFRTPSFMPVATNNASYYPQQQTP
LLYPGMEVCPHDKSTAQPPATTTMYLQAPPSSAHLAAAAGRGAAEAEGHGRRGGGAGGRE
TLQLFPLQPTFVLPDHKPLRAGSACAAVSPTTPSASASFSWESESSDSPSSEAPPFYDFFGVHS
GGR
>LOC_Os01g63510.1
MEALSGRVGVKCGRWNPTAEQVKVLTELFRAGLRTPSTEQIQRISTHLSAFGKVESKNVFY
WFQNHKARERHHHKKRRRGASSPDSGSNDDDGRAAAHEGDADLVLQPPESKREARSYGH
HHRLMTCYVRDVVETEAMWERPTREVETLELFPLKSYDLEVDKVRYVRGGGGEQCREISFF
DVAAGRDPPLELRLCSFGL
>LOC_Os03g20910.1
MEGSSNSPDRQSSGGSPPEERGGGGSGGGGGRSAAGEPVRSRWTPKPEQILILESIFNSGMVN
PPKDETVRIRKLLERFGAVGDANVFYWFQNRRSRSRRRQRQMQAAAAAAAAAASSSSPSA
NTSPAAASAATVQVGLPPGAVVHTMAMGGSACQYEQQASSSSSSGSTGGSSLGLFAHGAG
ASGAGGYLQASCGASASASSALAPGLMGDVVDSGGSDDLFAISRQMGFVGSPRCSPASSPA
TPSSAATAAQQQFYSCQLPAATITVFINGVPMEMPRGPIDLRAMFGQDVMLVHSTGALLPVN
DYGILMQSLQIGESYFLVTIHLQALTSWSHRSLSTPISQCSFVFMKAPHF
>LOC_Os04g56780.1
MDHMQQQQRQQVGGGGGEEVAGRGGVPVCRPSGTRWTPTTEQIKILRELYYSCGIRSPNSE
QIQRIAAMLRQYGRIEGKNVFYWFQNHKARERQKKRLTTLDVTTTTAAAADADASHLAVL
SLSPTAAGATAPSFPGFYVGNGGAVQTDQANVVNWDCTAMAAEKTFLQDYMGVSGVGCA
AGAAPTPWAMTTTTREPETLPLFPVVFVGGDGAHRHAVHGGFPSNFQRWGSAAATSNTITV
QQHLQQHNFYSSSSSQLHSQDGPAAGTSLELTLSSYYCSCSPYPAGSM
>LOC_Os04g55590.1
MRLHHLHVAYLDHKASSSSSSPAPPSISPSSIPGSAAFPAFSFKCLRPLAPKISLPEPRKMIAPP
DFVVPRARNASKLLNYTVQVPAAGTTRWNPSAEQIKVLEMLYRGGMRTPNSVQIERITEEL
GKYGRIEGKNVFYWFQNHKARERQKQKRAALLTLSTLDPSLLPATANETKEAPEKKEKDVE
DGLASCKRRCKAWGDGAGDGDAVVATEAAGGCTDEVTLELFPLHPQGKA
>LOC_Os05g02730.1

MAPAVQQQQSGGGGGSTGAAAVGSTTRWCPTPEQLMMLEEMYRGGLRTPNAAQIQQITAH
LSTYGRIEGKNVFYWFQNHKARDRQKLRRRLCISHHLLSCAHYYHHHLAAAAAVVPPPQLL
PPLHPSSSSSSCGGGLIDHANSLLSPTSATTPTSAAAAAAAAAYTTSYYYPFTAAAAPPPPRTS
PAASPLFHYNQGGGGVVLPAAEAIGRSSSSSDYSLGKLVDNFGVALEETFPAQPQQPATTMA
MTAVVDTTAVAAAAGGFCRPLKTLDLFPGGLKEEQHDVV
>LOC_Os05g48990.1
MASPNRHWPSMFRSNLACNIQQQQQPDMNGNGSSSSSFLLSPPTAATTGNGKPSLLSSGCEE
GTRNPEPKPRWNPRPEQIRILEGIFNSGMVNPPRDEIRRIRLQLQEYGQVGDANVFYWFQNR
KSRTKNKLRAAGHHHHHGRAAALPRASAPPSTNIVLPSAAAAAPLTPPRRHLLAATSSSSSSS
DRSSGSSKSVKPAAAALLTSAAIDLFSPAPAPTTQLPACQLYYHSHPTPLARDDQLITSPESSS
LLLQWPASQYMPATELGGVLGSSSHTQTPAAITTHPSTISPSVLLGLCNEALGQHQQETMDD
MMITCSNPSKVFDHHSMDDMSCTDAVSAVNRDDEKARLGLLHYGIGVTAAANPAPHHHHH
HHHLASPVHDAVSAADASTAAMILPFTTTAAATPSNVVATSSALADQLQGLLDAGLLQGGA
APPPPSATVVAVSRDDETMCTKTTSYSFPATMHLNVKMFGEAAVLVRYSGEPVLVDDSGVT
VEPLQQGATYYVLVSEEAVH
>LOC_Os07g34880.1
MMALGVPPPPSRAYVSGPLRDDDTFGGDRVRRRRRWLKEQCPAIIVHGGGRRGGVGHRAL
AAGVSKMRLPALNAATHRIPSTSPLSIPQTLTITRDPPYPMLPRSHGHRTGGGGFSLKSSPFSS
VGEERVPDPKPRRNPRPEQIRILEAIFNSGMVNPPRDEIPRIRMQLQEYGQVGDANVFYWFQ
NRKSRSKNKLRSGGTGRAGLGLGGNRASEPPAAATAHREAVAPSFTPPPILPPQPVQPQQQL
VSPVAAPTSLSSSSSDRSSGSSKPARATLTQAMSVTAAMDLLSPLRRSARPRQEQRHV
>LOC_Os07g48560.1
MDGGHSPDRHAAAAAGEPVRSRWTPKPEQILILESIFNSGMVNPPKDETVRIRKLLERFGAV
GDANVFYWFQNRRSRSRRRQRQLQAQAQAAAAAASSGSPPTASSGGLAPGHAGSPASSLG
MFAHGAAGYSSSSSSSWPSSPPSVGMMMGDVDYGGGGDDLFAISRQMGYMDGGGGSSSSA
AAGQHQQQQLYYSCQPATMTVFINGVATEVPRGPIDLRSMFGQDVMLVHSTGALLPANEY
GILLHSLQMGESYFLVTRSS
>LOC_Os08g14400.1
MDRTATASWEVMSRRGEQQQQLMMQAPASHNGGSGGGEPARSRWAPKPEQILILESIFNSG
MVNPAKDETARIRRLLERFGAVRDANVFYWFQNRRSRSRRRARQLQQACGAALHQLPSAA
AAAGAGGGGDYYHHHHQPSSSPFLMHGGGGGGVVTSTTAAPAVAASGHFLADEVDGGGD
DDLFAISRQMGLMARHGGGDHHYSSYADSDATQLSYQPTGTIQVFINGVAYDVPSGGALD
MAGTFGRDAMLVHSSGEVLPVDEHGVLINSLQMGECYYLVSKSI
>LOC_Os11g01130.1
MPQTPSTRWCPTPEQLMILEEMYRSGVRTPNAAEIQQITAHLAYYGRIEGKNVFYWFQNHK
ARERQRLRRRLCARHQQQPSPPSSTVPPAPTAAAAGAVVQVHPAVMQLHHHHHHHHPYAA
AAAAQSHHLQQQQQQQAEWPAAVDYCSTASASASATAADMAIPPCCRPLKTLELFPTKSTS
GGLKEDCCSSSKSSSCSTSTN
>LOC_Os12g01120.1
MPQTPSTRWCPTPEQLMILEEMYRSGVRTPNAAEIQQITAHLAYYGRIEGKNVFYWFQNHK
ARERQRLRRRLCARHQQQPSPPSSTVPPAPTAAAAGAVVQVHPAVMQLHHHHHHHHPYAA
AAAAQSHHLQQQQQQQAEWPAAVDYCSTASASASATAADMAIPPCCRPLKTLELFPTKSTS
GGLKEDCCSSSKSSSCSTSTN
>XP_031474977.1
MGGRNGGRMEGEELKQQQNGGGVGGNAGLYVKVMTDEQMELLRRQISVYATICEQLVEM
HKASTAQQDSVAGMRLGNIYADPSMAAAGHKVTTRQRWTPSQMQLQILESIFEQGNGTPSK
QKIKEITAELSQHGPISETNVYNWFQNRRARSKRKQQGTSANIESEVDTEVESPQEKRSKAEN

INFPENPALHVDGLSFRGQEVSSYMRSMDPQPSKMLHMQSNDDSKSSGNLTPFSFLENMLTN
PRMEHLMGKMEGPASFNPFHGNEGFDMIG
>XP_031475507.1
MEEGSGGGGELGRESSEQELHPTAAPLLHGRVDAQPLLLSLPLLVVLEPVEHSSLRIRICSAG
GAGGGSNKSSFLCRQSSTRWIPTAEQIRILRELYYNNGVRSPSAEQIQKISARLRQYGKIEGKN
VFYWFQNHKARERQKKRLSTDAAVQQRCSAVGWSSCSDDFSPKFTSSSTSLLHTSAPGISSC
GASIVSGGXMAGVSSALVERGSFG
>XP_031478028.1
MESQNQIQQEDGNGGGGAGGGSNKSSFLCRQSSTRWIPTAEQIRILRELYYNNGVRSPSAEQI
QKISARLRQYGKIEGKNVFYWFQNHKARERQKKRLSTDAAVQQRCAAVGWSSCSDDFSPK
FTSSSTSLLHTSAPGISSCGASIVCGGQMTGVSSALVERGSFRDRYWEGGFCSGSTGGMTETS
ADTYGCFGQHDSDETHGADGDIETLQLFPLRGGENSEIGSDKPEADEFYPSWYFGDAGGEQ
GASLELTLGSFPTADGFLRGSGSAGIGGFCPGS
>XP_031479820.1
MKVQQLGTREPYSALCCKRPRPLMPKLSVSSSGLNHAENQPPAAPPPTVVEVHSGGTRWNP
TREQIGILEMLYRGGMRTPNAHQIESITAQLGRYGKIEGKNVFYWFQNHKARERQRQKRCS
VGLSLSTGSPQRDSLTPDSAKKYRGSLAGRPPFVDGDGAGRKQGEDKKLEEYSSYNKRKWR
SWGDQILEIESRAFGGQKQDKTLELFPLHPEANR
>XP_031486496.1
MPQPMSTRWCPTPQQLMILEDMYRAGIRTPKASQIQQITAHLSFYGKIEGKNVFYWFQNHK
ARERQKLRKRMMKELQHQYLLDRARDGAAGLHMQPHQYFFHHQQEGSRAMNFMCKVAT
PEVEEMRNPAAAAAAGGAGGIHEWVMLELSPPSCCRPLQTLELFPVTSTGLKDEKTATNSPT
STTTECSSSKTDE
>XP_031486648.1
MEEGLSEFCIRGGGGGGGVKCGRWNPTAEQVKVLTDLFKAGLRTPSAEQIQRISSQLSSYGK
IESKNVFYWFQNHKARERQKRRKISDKDDKKKIAAAAAAAAFFREADMSSTIDLLEQDSAV
RTLQLFPLKSYEKSDGLDRSPRLLTNGSTKETPPYSLFFEASIFSAATTTNTATAAAATRDPLD
LRLSSL
>XP_031487803.1
MEECKGDDGSATNVSGEVSGGGVSSRWNPTKEQISLLEGLYKQGIRTPSAEQIQQITGRLRV
YGHIEGKNVFYWFQNHKARQRQKQKQESLVYFNRFLHAAAIPFVPPLPPPNALAFLAISLFS
VISKEAHYGNMLPFFCLAVVCSPSYASHGGFGYYAPYQKVLLPAAGGARRPRAERSDAKIR
KQEQQGYGASDVGYGEGRSSLSGATSHETLQLFPLHPTGILCRDSGEEAFSSPSPVISSSDGNP
SCFSDDSDVSLSENTEQLPFINFFSQN
>XP_031487821.1
MPQAFSTRWCPTSEQLMILEDMYRGGIRTPNALQIQQITAYLSFYGKIEGKNVFYWFQNHKA
RERQKLRRKMSMHLHRHFHVDRAAANLGHVKQNQHFFPNHVQGTSRSVMNFACKLDNTG
RPEMRRPATVDGTGLKHEWVILELDPASYCRPLRTLDLFPVTSTGLKEEKTAGTAAAAATST
TTNPTACSCSKTTD
>XP_031493304.1
MGTMECGDTATSFNTTPDSCQTPRGLRPFIHRPQYPPSHVVHPSHPSSSSLSSTPTTTTKDILP
LNFFPTGKEQRRKDADPQSTVSSRWSPTAEQLRWLEDLYRRGIRTPTAEQIQYITAQLRRFG
KIEGKNVFYWFQNHKARERQKRRRRSEAAALELVLEIDNPDRNLDPPHEWLGELEKTKSWA
PTYRTNESQGDKWIGWDGELHKQQRQRRQQQQEHDSACGRTISAAAERVQSPSLVLQQP
QLCPPDGVVLQTGPSSAASLRARAQPGEGAAETSGRMGTRLTETLDLFPLHSDSDAPLKCSR
KERQEQRRFESDGTFTTMFLEEEHGGHGRRFFQFLPLKN
>XP_031495354.1

MVLRNGCGVMEGVEERQPENGGGGAAAASSGGGVYVKVMTDEQMELLRRQISVYATICE
QLVEMHKAITAQQDSIAGIRLGNMYCDPLMGGGHKITTRQRWTPSQMQLQMLENIFEQGN
GTPSKQKIKEITSELMQHGPISETNVYNWFQNRRARSKRKQQVGAPNNVESAAETEVESPKE
KKAKVENIHSHDKPTLHVDDMSYHGQEVSSNIYSLDPQSSKMQHTQSSDSLKSSGSLSPFSFF
ENMLANPRIEHLMGKMESQDFSPFHTNDGFDVIG
>XP_031495600.1
MLCGMESGEEIQQKDGSGDSSSNRSSYLCRQTSTRWVPTAEQIRILKELYYGNGLRSPTAEQI
QRISARLRQYGRIEGKNVFYWFQNHKARERQKKRLDADFAAQRTTAATTTATPVAVHRWS
SYGDFASKSTSILNTNVVVGISNATYLSFSVFHFPGLNTVATSCSGNVLSAGQVCRRSCGSVI
ANSGHRPDSSCLGRDGFISSGSSCTSETGTDSHGLFDPGDSGVAADRSAGILETLQLFPLRGS
DDSGTGSERSRIGETYPALCFDDASLELTLSSFPGGDGGMRNAGSGPTFLPSGS
>XP_031495601.1
MLCGMESGEEIQQKDGSGDSSSNRSSYLCRQTSTRWVPTAEQIRILKELYYGNGLRSPTAEQI
QRISARLRQYGRIEGKNVFYWFQNHKARERQKKRLDADFAAQRTTAATTTATPVAVHRWS
SYGDFASKSTSILNTNVVVGQVCRRSCGSVIANSGHRPDSSCLGRDGFISSGSSCTSETGTDSH
GLFDPGDSGVAADRSAGILETLQLFPLRGSDDSGTGSERSRIGETYPALCFDDASLELTLSSFP
GGDGGMRNAGSGPTFLPSGS
>XP_031495729.1
MNDGHQLADNGGSVGGAVRRDGCGVNQVAERTTGPIRSRWSPKPEQILILESIFNSGMVNPP
KDETVRIRKLLERFGSVGDANVFYWFQNRRSRSRRRLRQMQSGIQALIRDEKSSFHVDPAPF
CSSSSSSSSSSSFSPFSPPLLHDSVSPHLPQGNAAALGPTSGHGGLSVNDTYSASHLPVDEAVN
VYPVSKELGSHHIDQPYLVSSILTQSSSAGFHYQSELITVSINGIIFQVPRGPFHMRALFGEDVI
LVHSSGDPIHVDVSGVSLQGLQPGESYFLVNVPFPFPFRETVKFTNILFFAF
>XP_031497990.1
MWMMGCGDTASLNNTSDSCHATRRLRPIIPRPHLLHSSHHHSSSPSSTAADFLPLNISPSGIEQ
SGRRAEGSGAQQAVSSRWNPTPEQLRTLEELYRRGTRTPTAEQIQYITAQLRRFGKIEGKNV
FYWFQNHKARERQKRRRQLEAVTGVGREIESSDKKDSSYTSWNGYDGSKNLVASSCRGEE
RRVEDKWVRWDRELHDYSRQQPQQQQNLGGGCGRTIVAAAAAEREQAASALLLQRMQYS
SPPAGALLQSPRGATAVRAWAGGCDREEEVGEAGCGRETLELFPLRSDSSAVAARRCCHPQ
QQQRIHLENDSALMSSFVEEQQGARGRQFFQLPLKN

Supplementary Table S1 The primers for qRT-PCR of NnWOXs.

Gene	Primer	Sequence(5'to3')
NnWOX12	*NnWOX12*-F	GTGGGTTTAACATGGCGGAT
	NnWOX12-R	CGGTTACAGTGGCCAGATGA
NnWOX13	*NnWOX13*-F	TTCATCGACAGAGCAGTGGAC
	NnWOX13-R	GAGCTGCAGCGTTTCTATGG
NnWOX14	*NnWOX14*-F	GCAGGATCTCGCTGGAATGA
	NnWOX14-R	ATTTGGAGCTGGATGGGTGT
NnWOX15	*NnWOX15*-F	CTGTCAGCTTGAGTCACTTGTC
	NnWOX15-R	GATTAAAGCTCCCTGGGCCT
NnACT	*NnACT*-F	TGGCAGACAACGAGGATATTC
	NnACT-R	CTACAATGCTAGGGAACACGG

Supplementary Table S2. The Seqlogo of motifs.

Motif	The Seqlogo of motif
Motif 1	ILEEJYRSGMRTPSADQIQKITAQLSFYGKIEGKNVFYWFQNHKARERQK
Motif 2	PTASTRWNPTPEQJR
Motif 3	AFEVAVGPLNVREAFGEDAILVHSSGLPVJTNEWGVTLQSLQHGAFYYLV
Motif 4	EEEREIETLZLFPLHSE
Motif 5	MYVKVMTDEQMELLRKQISIYATICEZLVEMHKAITAQRDLAGMRLGNLY
Motif 6	MWMMGCSDGSGFNMPDSFNGRKLRPLMPRLTTNSTNSAAGIAPCLSRIHG
Motif 7	LNMKVHQLARGLWEHEPSLTLGCKRLRPLVPKLTNGESPANLDLKSFIKP
Motif 8	EETVSMQRAAVAESRTDGWIQFEEGELQQRRSSVERNPTWQMMQL
Motif 9	EIEREEDSPYEKKHKPWAIEFHEND
Motif 10	WMYGRDWMMMMMD
Motif 11	DQLTWPCPGGLLLNDJINQGAPKKDDQVKMK
Motif 12	MDERMRGFCIRAGGGGYGGGG
Motif 13	EKSPPRRTDKPPPAGGDNMIEASNSPTASVNQTYFQPQLD
Motif 14	DEEKETEVPNPTMNSNFTSYQFFEFLPMK
Motif 15	AEDLCFQSPEIISELHSLDPQPNKAEAIFPLDGTSKSSGSLSHLSFYESV

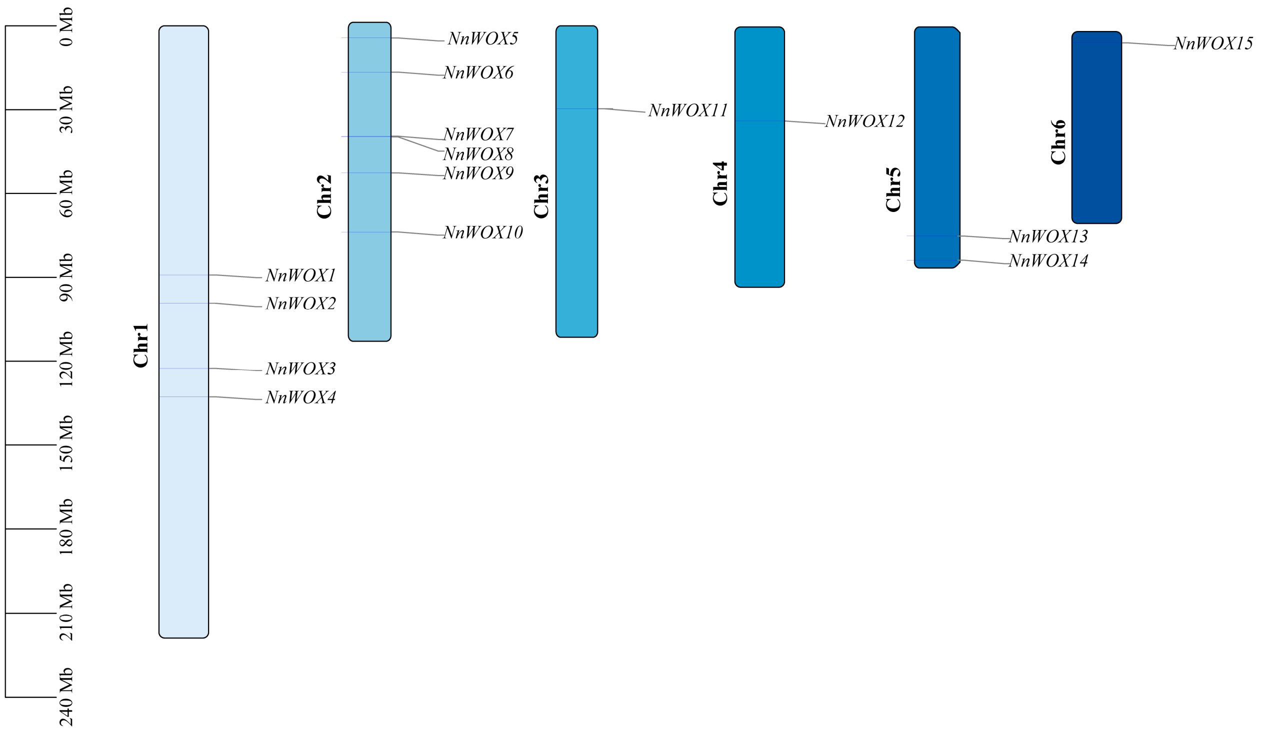

Supplementary Figure S1. Distribution of NnWOXs on chromosomes.

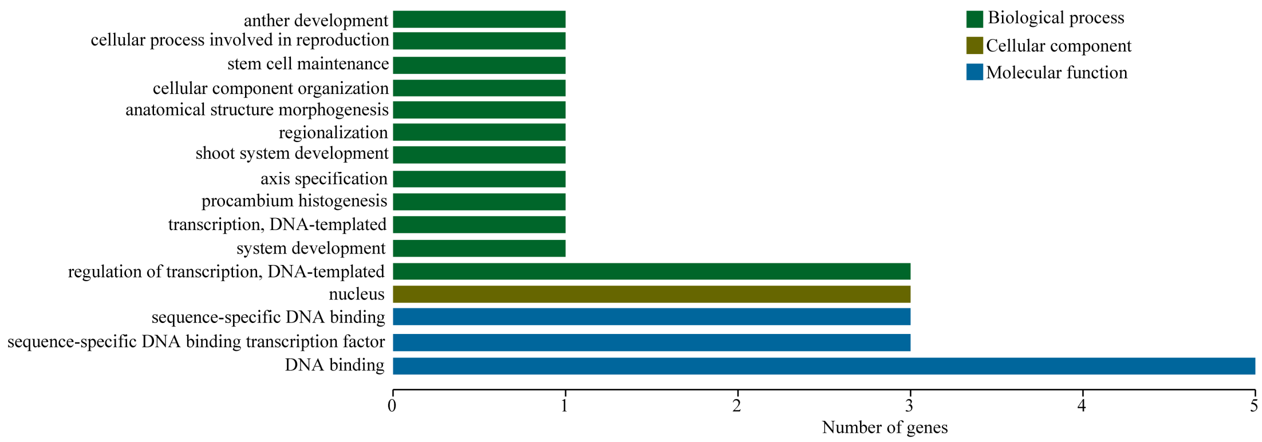

Supplementary Figure S2. The Go annotation of NnWOX gene family.
